# Replay of procedural memory is independent of the hippocampus

**DOI:** 10.1038/s41593-026-02362-5

**Published:** 2026-07-23

**Authors:** Emmett J. Thompson, Lars B. Rollik, Benjamin Waked, Georgina Mills, Sthitapranjya Pati, Jasvin Kaur, Ben Geva, Haoyu Li, Rodrigo Carrasco-Davis, Tom George, Clementine Domine, William Dorrell, Marcus Stephenson-Jones

**Affiliations:** 1 Sainsbury Wellcome Centre for Neural Circuits and Behaviour, University College London, London, UK; 2 Gatsby Computational Neuroscience Unit, University College London, London, UK

**Keywords:** Consolidation, Hippocampus

## Abstract

Sleep is crucial for consolidating all forms of memory and a core mechanism underlying this process is offline replay. Current models propose that replay originates in the hippocampus and triggers reactivation across cortical and subcortical networks. However, conflicting evidence about the role of the hippocampus in offline consolidation of nondeclarative memories raises the question of whether hippocampal replay drives their consolidation. Here we show that replay occurs in the dorsal striatum during offline consolidation of a procedural memory in mice, independently of the hippocampus, and that its content predicts subsequent performance improvements. Neural sequences linked to salient behavioral events were prioritized for replay, with positive and negative behavioral outcomes having opposing effects on individual replay events. All features of replay persisted despite complete bilateral hippocampal lesions. These findings demonstrate that procedural replay occurs independently of the hippocampus, indicating that replay-driven memory consolidation can operate through parallel, independent mechanisms.

## Abstract (web summary)

Thompson, Rollik and colleagues show that, during sleep, the brain replays procedural memories in the striatum independently of the hippocampus. Replay content was shaped by prior reward and error outcomes and predicted next-day motor performance improvements.

## Main

The hippocampus is thought to be crucial for the sleep-dependent consolidation of both declarative memory and memories that do not require the hippocampus for their expression, such as procedural memory^1–8^. During sleep, hippocampal replay of spatiotemporal context is thought to trigger the active consolidation of all kinds of memory by boosting context-related representations throughout cortical and subcortical circuits^1–3,5,6,9–14^. Under this ‘systems consolidation’ framework, the hippocampus acts as a global orchestrator of offline reactivation—even for memories ultimately stored in nonhippocampal circuits, such as the procedural memories encoded in corticostriatal circuits. Consistent with this view, several studies have reported hippocampal involvement in the sleep-dependent consolidation of nondeclarative memories, including procedural skills^6–8,11,15^.

However, whether the hippocampus plays an obligatory role in the consolidation of nondeclarative memories is a matter of ongoing debate^6,8,11,13,15–23^. Indeed, hippocampal lesions can facilitate rather than impair procedural and associative learning^17,20,22,23^. This creates a fundamental and unresolved tension at the heart of systems consolidation theory: if a memory is encoded and expressed independently of the hippocampus, must its sleep-dependent consolidation nevertheless depend on hippocampal replay or do nondeclarative memory circuits possess an autonomous capacity to generate replay?

Here we address this question directly. We show that replay of procedural memory in the dorsal striatum occurs independently of the hippocampus during offline consolidation. These findings suggest that replay is a conserved mechanism for memory consolidation that operates through parallel, autonomous processes across distinct memory systems, challenging the view that the hippocampus acts as its universal orchestrator.

## Results

### DLS is required for a multistep procedural memory task

**Fig. 1:**
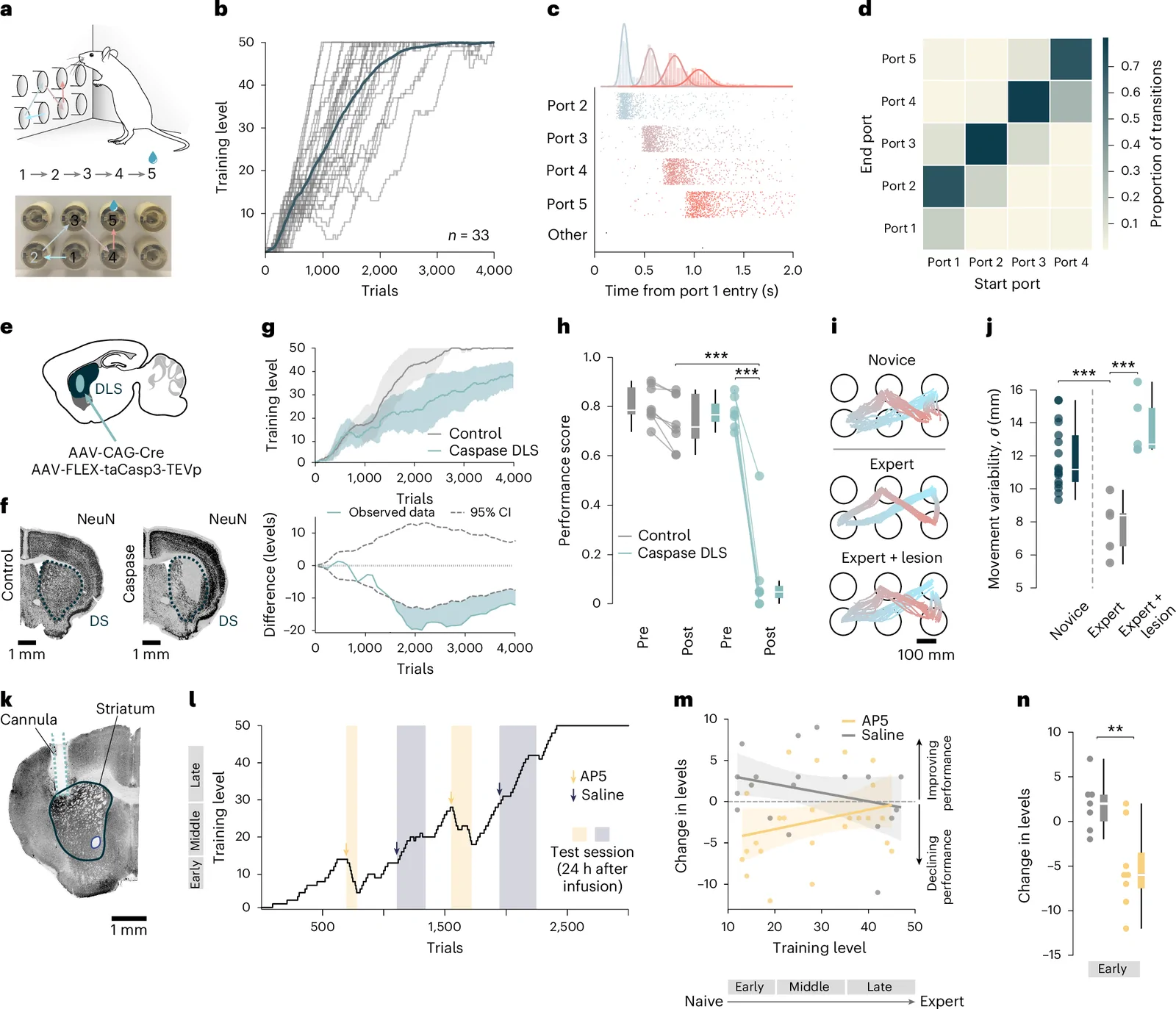
Learning and execution of the sequence learning task is dependent on the DLS. **a**, Top: schematic of the task. Bottom: photograph of the port wall with task sequence overlaid. **b**, Training level progression curves (gray) for multiple animals with mean learning curve overlaid (dark green) (*n* = 33 animals, mean trials to level 50 = 1,942 ± 111 (s.e.m.)). **c**, Example poke times from an expert animal. Port-poke-in times are shown (points) for each port relative to the start of trial initiation. All pokes in task-irrelevant ports are labeled ‘other’. **d**, Transition heatmap showing mean port-to-port transition proportions for multiple trained animals (*n* = 33). Each transition in the task sequence is represented by start (*x* axis) and end (*y* axis) ports (normalized by column). **e**, Schematic of the experimental approach for bilateral lesions of the DLS. **f**, Histology: coronal sections showing neurons in one example hemisphere of the striatum (dark blue outline) for lesion and control mice. **g**, Top: learning curve (training level versus trials) for control and lesion animal groups (line is mean; shaded area denotes s.d.; lesion *n* = 7 mice; control, *n* = 6 mice). Bottom: differences in performance between the groups. Dotted lines indicate the 95% confidence interval (CI) for the shuffled data (Methods). **h**, Average task performance scores (Methods) for the three sessions pre-injection and post-injection surgery (2 × 2 two-way analysis of variance (ANOVA); control versus lesion, ^***^P < 0.001, *F*-statistic (*F*) = 61.9, eta-squared (*η*^2^) = 0.3; pre versus post, ^***^P < 0.001, *F* = 68.9, *η*^2^ = 0.3; group versus surgery interaction, ^***^P < 0.001, *F* = 50.1, *η*^2^ = 0.2; two-way permutation ANOVA confirmed all effects, ^***^P < 0.001; post hoc Tukey honestly significant difference (HSD); control pre versus post, *P* = 0.6164, Cohen’s *d* = 0.76; lesion pre versus post, ^***^P < 0.001, Cohen’s *d* = 4.87; control post versus lesion post, ^***^P < 0.001, Cohen’s *d* = 4.26; lesion, *n* = 7 mice; control, *n* = 8 mice). **i**, Movement tracking (animal head) for four subsequence movement vectors, for example, novice, expert and lesioned expert mouse. **j**, Average movement variability (s.d. from mean trajectory) across the four subsequence trajectories for novice mice (*n* = 8 mice, *n* = 18 sessions) and expert mice (*n* = 5 mice) before and after a lesion to the DLS (one-way ANOVA; ^***^P < 0.001, *η*^2^ = 0.65; novice versus expert, Tukey HSD, ***P < 0.001, Cohen’s *d* = 2.67; novice versus lesion, Tukey HSD, ^***^P < 0.001, Cohen’s *d* = 0.80). **k**, Histology showing an example hemisphere of a coronal slice after bilateral cannula implantation. Cannula tract (light blue) and striatum (dark blue) are outlined. **l**, Example animal learning curve showing AP5 and saline infusions (arrows) and test sessions 24 h after infusion (shaded). Infusions were performed the day before the test session, immediately after training that day (Methods). **m**, Training level changes against the start level for all post-infusion test sessions (saline and AP5) for all animals (*n* = 8). The lines show linear regression fit for each dataset with CI (shaded region) (categorical interaction coefficient, ordinary least squares regression (OLS); ^*^P = 0.01, *R*^2^ = 0.20). **n**, Training level change for early learning infusion experiments (two-sided, independent Student’s *t*-test; ^**^P = 0.005, Cohen’s *d* = 1.78; *n* = 7 animals, saline *n* = 7 sessions, AP5 *n* = 8 sessions). Boxplots show the median, interquartile range (IQR) and range. ^*^P < 0.05, ^**^P < 0.01, ^***^P < 0.001. DS, dorsal striatum. Mouse drawing in **a** adapted from Scidraw under a Creative Commons license CC BY 4.0. Source data

**Extended Data Fig. 1:**
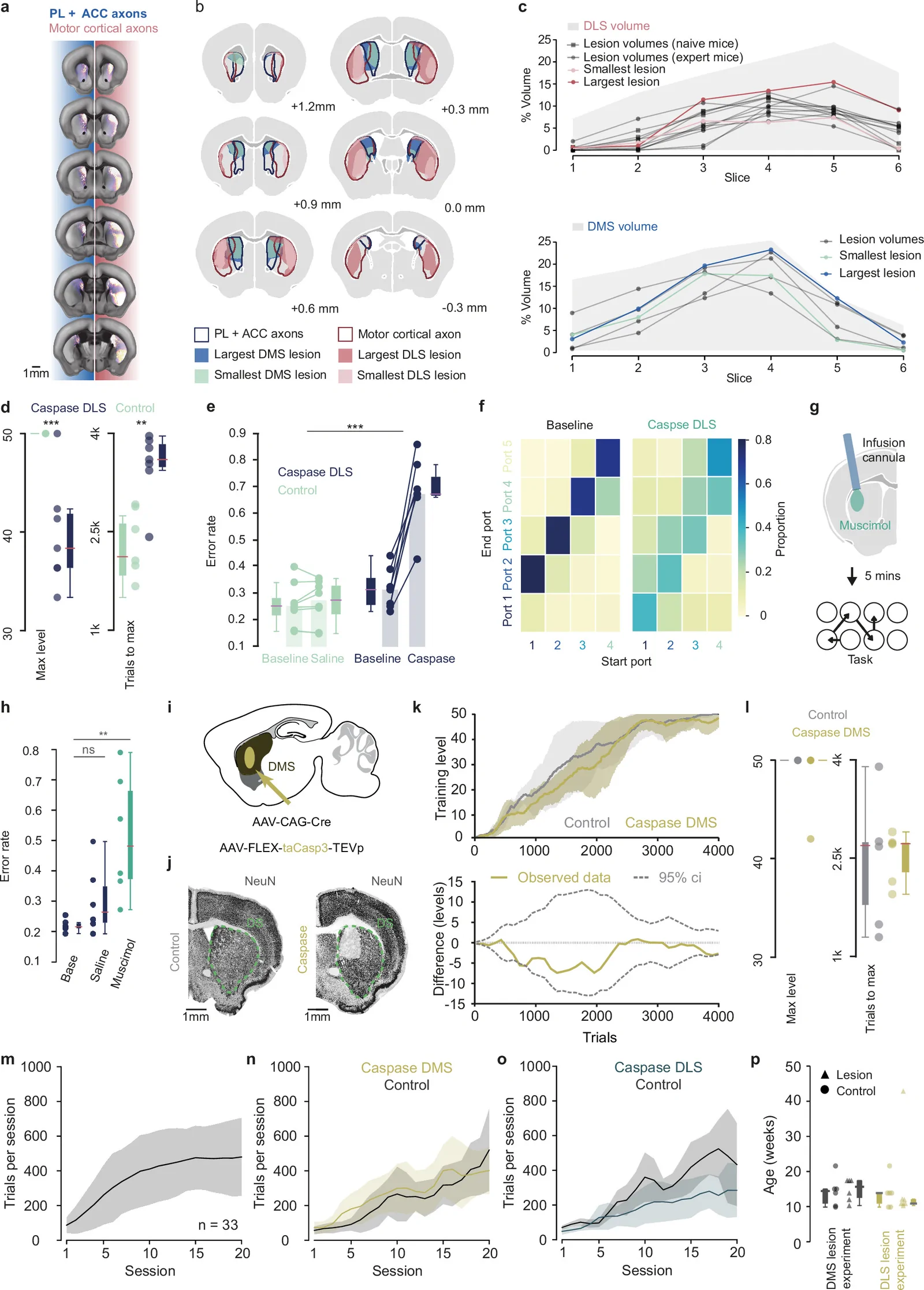
Histology and quantification for striatal lesions. **a**. Allen reference projections from prelimbic and cingulate cortex (left, Allen experiment: 157711748 & 112514202) and motor cortex (right, Allen experiment: 180720175 & 180709942). **b**. Allen projection defined DMS (blue line) with largest DMS lesion (blue shaded) and smallest DMS lesion (turquoise shaded) for the extent of striatum. Allen projection defined DLS (red line) with largest DLS lesion (red shaded) and smallest DLS lesion (pink shaded) for the extent of striatum. **c**. Percentage of total DLS and DMS volumes lesioned for each animal in the two respective groups. Total volume of each region is shown (grey background) as well as the smallest and largest lesions in each group (light and dark coloured lines). DLS lesions are further subdivided into those animals lesioned while naive; for pre-training experiments (square markers), and animals that were lesioned while task experts for post-training experiments (circle markers). **d**. Left: maximum training level achieved within criterion (4000 trials) for DLS lesion and control animals. Right: trials to reach maximum attained level (See panel l. for statistics). Lesion, n = 7 mice. Control, n = 6 mice). **e**. For DLS lesions and controls, proportion of port-to-port transition errors in the 3 sessions before and 3 sessions after injection surgery (Pairwise pre/post differences between groups; MannWhitney U; ***p < 0.001, Rank-Biserial r = 1. **f**. Mean transition histograms before and after injection surgery**. g**. Schematic showing experimental design for bilateral muscimol infusions. **h**. Port-to-port transition error rates for baseline sessions, saline infusions and muscimol infusions (repeated measures one-way ANOVA; **p < 0.007, η^2^ = 0.49. Tukey HSD, Cohens’s d; p = 0.558, d = 1.01, **p = 0.006, d = 1.96. n = 6 mice). **i**. Schematic of the experimental approach for bilateral lesion of the DMS. **j**. Coronal sections showing neurons in one example hemisphere of the striatum (green outline) for DMS lesion and control mice. **k**. Top: Learning rate (training level vs trials) progression curves for control and lesion animal groups (shaded area denotes standard deviation). Bottom: differences in performance between the groups. Dotted lines indicate the 95% confidence interval for the shuffled data (see methods). **l**. Left: maximum training level achieved within criterion (4000 trials) for DMS lesion and control animals. Right: trials to reach maximum attained level (Comparative statistics with DLS experiment in panel d. Max level achieved, DLS vs DMS, two-way ANOVA; **p = 0.003, η^2^ = 0.36. DLS, lesion vs control, Tukey HSD; ***p < 0.001, Cohen’s d = 2.61. DMS, lesion vs control, Tukey HSD; p = 0.896, Cohen’s d = 0.58. DLS lesion vs DMS lesion, Tukey HSD; ***p < 0.001, Cohen’s d = 1.99. Trials to max; DLS vs DMS, two-way ANOVA; *p = 0.022, η^2^ = 0.22. DLS, lesion vs control, Tukey HSD; **p = 0.007, Cohen’s d = 2.40. DMS, lesion vs control, Tukey HSD; p = 0.999, Cohen’s d = 0.08. DLS lesion vs DMS lesion, Tukey HSD; p = 0.061, Cohen’s d = 1.91. DMS lesion; n = 6 mice, control; n = 6 mice **m**. Mean number of trials performed per session over the course of 20 training days (shaded area is standard deviation, n = 33 mice). **n**. Same as m. but for control and DMS lesion group mice. **o**. Same as m. but for control and DLS lesion group mice. **p**. Ages for control and lesion group mice for each learning related lesion experiment performed (DMS, two-sided independent t-test; p = 0.808, Cohen’s d = 0.15,. DLS, Wilcoxon rank-sum; p = 0.718, Rank-Biserial r = 0.14, DLS Lesion, n = 7 mice. Control, n = 6 mice. DMS lesion; n = 6 mice, control; n = 6 mice). Boxplots show median, IQR and range up to 1.5 IQR. Displayed stars: *p < 0.05, **p < 0.01, ***p < 0.001. Source data

To investigate the mechanisms that support offline consolidation of procedural memory, we developed a multistep procedural memory task for mice. Mice were rewarded for nose-poking in the correct sequence of five ports (Fig. 1a, Supplementary Fig. 1a,b and Supplementary Video 1). Mice learnt to produce the sequence from memory (Fig. 1b) and once experts completed the full task with highly stereotyped timing and accuracy (Fig. 1c,d and Supplementary Fig. 1c). Trial-to-trial movement variability decreased across learning (Supplementary Fig. 1d), consistent with the notion that animals had acquired a stereotyped procedural skill. Motor skill learning and execution is thought to depend on the dorsolateral striatum (DLS)^24–26^. To confirm that the DLS was important for learning and executing our multistep task, we lesioned this structure before training using a viral caspase strategy (Fig. 1e,f and Extended Data Fig. 1a–c). Compared to control mice, animals with bilateral DLS lesions showed impaired task learning (Fig. 1g and Extended Data Fig. 1d). The learning curves between groups diverged at a training level where visual guidance was removed, indicating that the DLS is needed for learning to perform the sequence from memory but not in a light-guided manner. Post-learning, DLS lesions or inactivation impaired task performance and increased movement variability (Fig. 1h–j and Extended Data Fig. 1e–h). In contrast, we were unable to detect any difference between lesion and control mice when we lesioned the adjacent dorsomedial striatum (DMS) (Extended Data Fig. 1i–p). Taken together, these results show that the DLS is required for learning and executing the motor sequence from memory.

**Extended Data Fig. 2:**
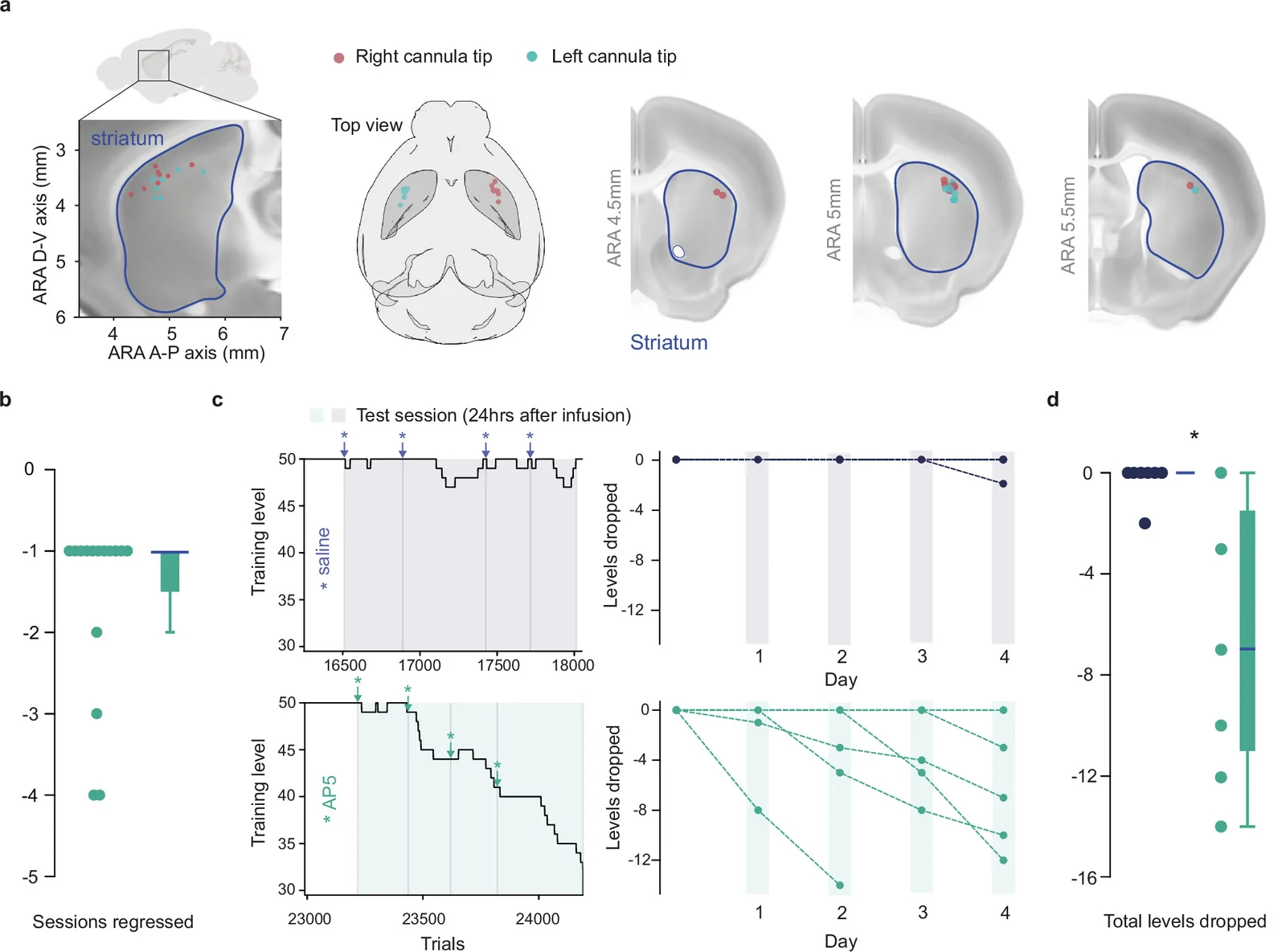
Blocking offline plasticity in the DLS impairs late stabilisation of procedural memory. **a**. Left and right hemisphere cannula tip positions in Allen reference atlas coordinates. left to right: Sagittal view, top-down view, and coronal sections. **b**. Number of sessions since minimum level was last seen for AP5 infusions experiments that resulted in negative trial changes (n = 7 animals; 14 sessions). **c**. Left: example training level progression for fully trained animals given consecutive infusions (infusions points marked by stars, sessions are marked by grey vertical lines, shaded regions are test sessions – 24 h post infusion). Right: cumulative levels dropped across the four consecutive infusion days for each animal. **d**. Summary plot showing total levels decreased for each infusion type (AP5 & saline) after 4 days of consecutive post session infusion (two-sided Mann-Whitney U test; *p = 0.024, Rank-Biserial r = 0.67. n = 6 mice). Boxplots show median, interquartile range and 1.5 IQR. Displayed stars: *p < 0.05. Source data

To determine whether the DLS is involved in offline consolidation, we infused the *N*-methyl-D-aspartate (NMDA) receptor antagonist 2-amino-5-phosphonopentanoate (AP5) into the DLS to disrupt offline striatal activity and plasticity^27–29^. This was done immediately after the days of training, before placing mice back into their home cage to sleep (Fig. 1k,l and Extended Data Fig. 2a). Testing the next day, 24 h after infusion, across all infusion sessions, there was a significant effect of AP5 infusion on performance (Fig. 1m). In early training, mice in the AP5 cohort were significantly worse at the task the following day (Fig. 1n), dropping on average to a training level that they had been at the day before the infusion session (Extended Data Fig. 2b). This suggests that, in line with current literature^28,30^, offline NMDA-dependent plasticity in the DLS is critical for consolidating the previous day’s performance gains. To determine whether offline activity and plasticity were also critical for maintaining procedural memory, we infused saline or AP5 for 4 d consecutively in the DLS post-training (Extended Data Fig. 2c). Infusion of AP5 for 4 d consecutively led to a significant drop in performance (Extended Data Fig. 2d). Together these data suggest that offline processes in the DLS are critical for both learning and maintaining procedural memory of a stereotyped action sequence.

### An unsupervised approach for replay detection

**Fig. 2:**
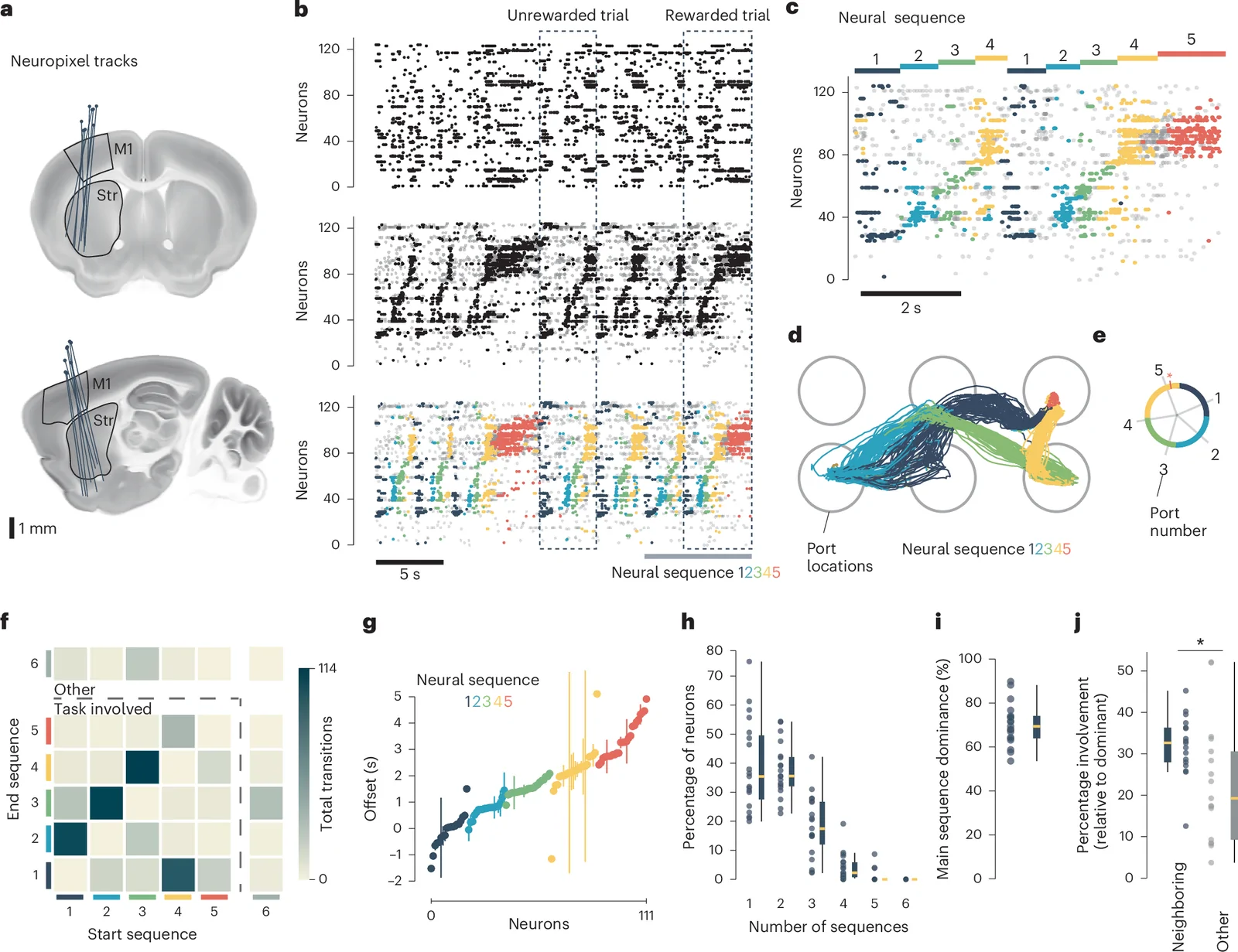
Unsupervised labeling of latent neural sequences in DLS during task practice. **a**, Schematic showing implanted Neuropixel probe locations projected onto standard Allen Atlas coronal (top) and sagittal (bottom). Primary motor area (M1) and the striatum (Str) are highlighted. **b**, Top: unordered example spike raster showing spikes for neurons in striatum recorded during task execution. Middle: the same spike raster but with neurons ordered lexicographically based on a PP-Seq-identified neural structure. Bottom: same as above but spikes colored by the latent PP-Seq sequence to which they were attributed. A rewarded and an unrewarded trial are shown (dashed boxes). **c**, Example PP-Seq-ordered spike raster (as in **b**, gray bar, bottom right) zoom in, showing two task trials with task-relevant sequences labeled. **d**, Example movement tracking (animal head) from 400 s of task engagement, colored by the current dominant PP-Seq sequence. **e**, Circularized task space colored by the dominant PP-Seq-labeled neural sequence. The hidden (nondominant on average) task-relevant sequence is represented by a star. Gray lines indicate respective task port locations across standardized space. **f**, Transition histogram showing numbers of sequence–sequence transitions during the example epoch. Task-associated and nontask-associated sequences are separated by the dotted line. **g**, Mean spike times during instances of each neuron’s dominant PP-Seq sequence for the example animal (error bars show the s.d. over sequence instances; *n* = 111 neurons). Neurons are ordered by offset from the earliest neuron in each sequence and sequences by mean distance between sequence midpoints. **h**, Number of sequences in which neurons appeared. For each analyzed recording session, the mean percentage of neurons that contribute (appearing at least 10% of the time) to different numbers of sequences is shown (*n* = 21 sessions, *n* = 8 mice). **i**, Mean relative percentage per session of spikes that were present in their most common (dominant) sequence (*n* = 21 sessions, *n* = 8 mice). **j**, Mean neuron occurrences between neighboring and distal sequences for neurons in each analyzed recording session. Mean proportions are calculated relative to the dominant sequence proportion and normalized by the number of sequences. The sequence order was the most observed task order and neighboring sequences were defined by those adjacent to the dominant (two-sided, paired Student’s *t*-test; ^*^P = 0.027, Cohen’s *d* = 0.59; *n* = 21 sessions, *n* = 8 mice). Boxplots show the median, IQR and range. ^*^P < 0.05. Source data

If an offline process supports procedural memory formation in the DLS, what could the underlying mechanism be? For episodic memories, replay of previously observed neural activity is thought to be the way in which memory is consolidated offline^9,10^. To search for similar neural reactivations in the DLS we implanted Neuropixel probes through this region and recorded neural activity during task execution and post-task sleep (Fig. 2a). Typically replay has been identified using template-based approaches such as template matching or Bayesian decoding^12,14,31,32^. These approaches often carry assumptions, such as that neural sequences progress in a linear fashion, in a constant direction at a constant speed. These assumptions have been shown to limit the types of replay that have been detected in the hippocampus^33^. In the hippocampus, these methods are also usually applied during candidate epochs defined by the presence of sharp-wave ripple (SW-R) oscillations, which are thought to be a marker for hippocampal replay^34^. As we did not know of a heuristic biomarker for replay in the striatum and did not want to, a priori, limit the types of reactivations that we could detect, we adapted an unsupervised point process model called PP-Seq^35^.

PP-Seq aims to attribute individual neuronal spikes to a latent cause, in this case instances of particular neural sequences. Via an iterative collapsed Gibbs’ sampling procedure, the model fits free parameters to determine the number of latent events (neural sequences) and then attributes spikes to these sequences based on features of activity (Supplementary Fig. 2a). In our raw Neuropixel data, there were no visible sequential patterns; however, clear sequences were revealed by sorting the neurons lexicographically according to the sequence type and the temporal offset parameter inferred by PP-Seq (Fig. 2b,c). Using a hyperparameter search, we found that our data were best described as containing six types of neural sequence (Supplementary Fig. 2b–f). To determine whether these neural sequences were related to aspects of our multistep task, we aligned these neural sequences with video-tracking data and colored the tracking by the dominant sequence type. Strikingly, despite the model having no access to behavioral information, different types of neural sequences aligned to distinct phases of the behavioral sequence (Fig. 2d–f and Supplementary Video 2). In every mouse recorded, the behavioral sequence was accompanied by multiple neural sequences that aligned to distinct phases of the task (Supplementary Fig. 2g,h). Not all neural sequences were aligned to distinct movements between the ports. In some animals, specific neural sequences correlated with reward consumption or aligned tightly to other nontask-related behavioral sequences that the mice performed during the recording session, such as grooming (Supplementary Fig. 2i and Supplementary Video 3). The neurons within a sequence type tended to spike at precise timing during the sequence and were also relatively exclusive, mostly spiking in one neural sequence type (Fig. 2g–i). When neurons did contribute spikes to multiple types of neural sequences, the spikes were most likely to occur in the preceding or following neural sequence (Fig. 2j). Taken together, applying PP-Seq revealed that there are multiple neural sequences in the striatum that occur at distinct phases of the behavioral sequence.

### Procedural replay in the DLS

**Fig. 3:**
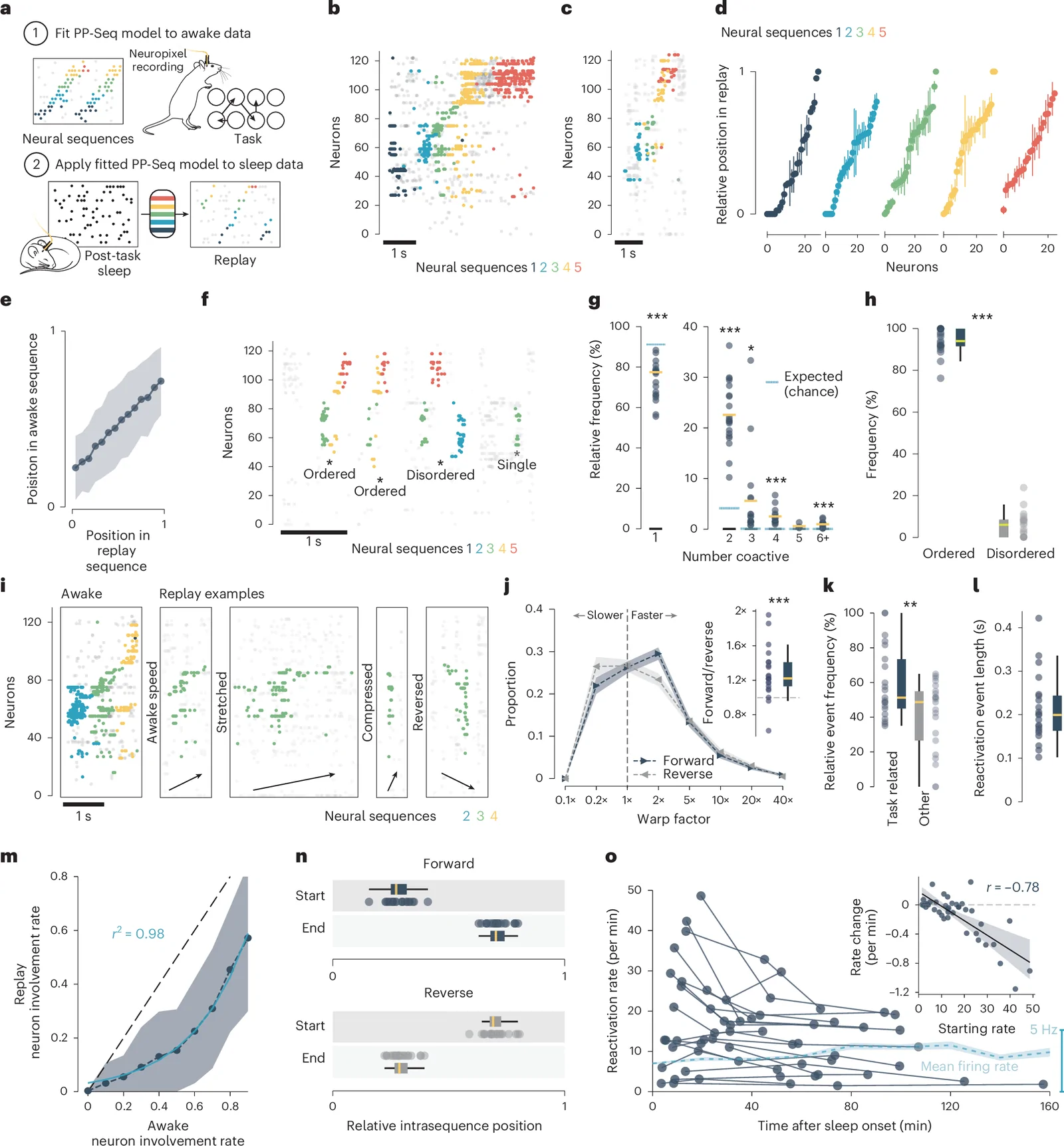
Replay of procedural neural activity in the striatum during sleep. **a**, Schematic of replay detection protocol: (1) PP-Seq model trained on and detects repeating neural sequences within task-related activity; and (2) fitted model applied to sleep recording to identify recapitulated task-related neural sequences. **b**, PP-Seq-labeled sequences for example task-related awake spikes. **c**, Example replay (same neurons as in **b**) of concatenated sequences that are ordered with respect to task-related activity. **d**, Mean relative position of spikes, for neurons in each replay sequence observed during the example recording. **e**, Mean relative positions in awake sequences versus replay sequences for all analyzed neurons across every session (linear relationship, OLS; correlation coefficient (*r*) = 0.63, coefficient of determination (*R*^2^) = 0.40, ^***^P < 0.001). **f**, Example sleep period with PP-Seq-labeled replay sequences. **g**, Frequency of single (isolated) and concatenated events for each recording session. (Difference from chance, two-sided, one-sample Student’s *t*-test, Cohen’s *d*: ^***^P < 0.001, *d* = −2.36; ^***^P < 0.001, *d* = 3.04; ^*^P = 0.044, *d* = 0.53; ^***^P < 0.001, *d* = 1.54; *P* = 0.098, *d* = 1.19; ^***^P < 0.001, 1.54.) **h**, Relative frequencies of task-ordered and task-disordered concatenated sequences (two-sided permutation test; ^***^P < 0.001, observed difference in means = 88.4%, 99th percentile permuted difference = 31.5%, Cohen’s *d* = 14.3). **i**, Example replay sequences with varied warp characteristics. **j**, Distribution of warp factors (1× represents awake speed) for forward and backward replay events (forward–reverse difference, PERMANOVA; *P* = 0.053, *R*^2^ = 0.74). Inset: proportion of forward versus reversed replays (1× represents equal proportion, difference from chance, two-sided, one-sample Student’s *t*-test: ^***^P < 0.001, Cohen’s *d* = 0.94). **k**, Normalized percentages of task-related and nontask-related replay observed (two-sided permutation test: ^**^P = 0.003, observed difference in means = 18.78%, 99th percentile permuted difference = 15.89%, Cohen’s *d* = 0.91). **l**, Average (mean) replay event lengths were observed for each recording session. **m**, Relative individual neuron involvement frequencies for each sequence during awake task activity and sleep periods (shaded area is the s.d., exponential function fit by nonlinear least squares; ^**^P < 0.001). **n**, Average (mean) start and end points for all forward (top) and reverse (bottom) replay events. The position is relative to the corresponding average awake sequence. **o**, Reactivation rates for each analyzed sleep epoch against time from first sleep onset (OLS; *r* = −0.23, *R*^2^ = 0.09, ^*^P = 0.019). The dashed line shows the mean firing rate for all neurons. Inset: the rate change against starting rate for each pair of analyzed epochs per session (OLS; *r* = −0.78, *R*^2^ = 0.61, ^***^P < 0.001). Boxplots show the median, IQR and range up to 1.5× IQR; shaded bands denote the s.e.m. unless stated otherwise; *n* = 21 sessions, *n* = 8 mice. ^*^P < 0.05, ^**^P < 0.01, ^***^P < 0.001. Mouse drawing in **a** adapted from Scidraw under a Creative Commons license CC BY 4.0. Source data

**Extended Data Fig. 3:**
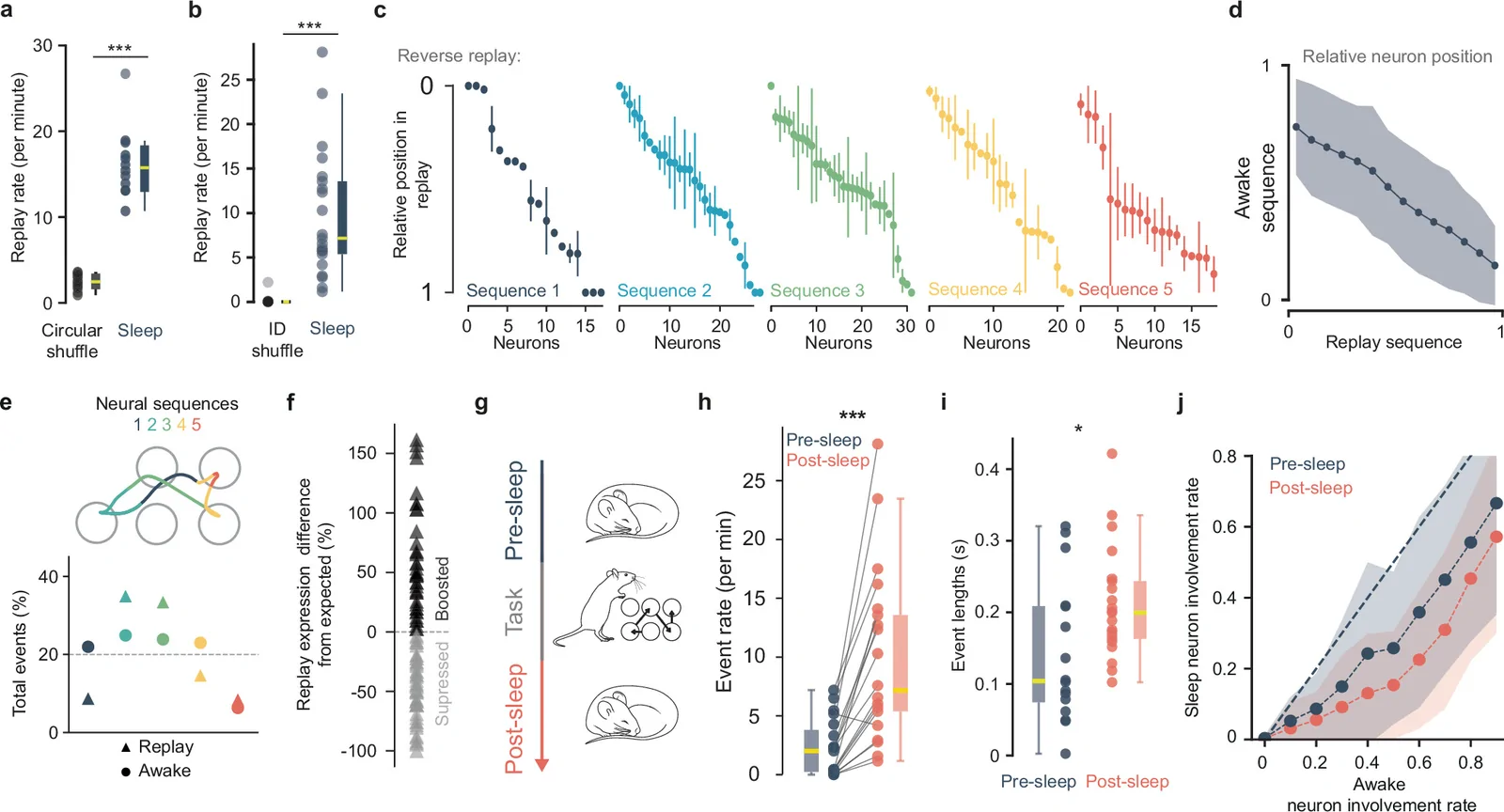
Features of striatal replay. **a**. Average (median) replay rate after circular shuffle (two-sided paired t-test; ***p < 0.001, Cohen’s d = 3.51, n = 12 mice). **b**. Average (median) replay rate per minute during sleep epochs and for shuffled data (neuron ID permuted, two-sided Wilcoxon rank sum, ***p < 0.001, Rank-Biserial r = 1. n = 21 sessions). **c**. Mean relative position of spikes, for neurons in each reversed replay sequence observed in example recording (error bars, SEM). **d**. Mean relative positions in awake sequences vs. reverse replay sequences for all analysed neurons across every session (shaded region = standard deviation, OLS regression, r = -0.58, ***p < 0.001, r^2^ = 0.40). **e**. Top: example animal’s average movement tracking during task execution coloured by the dominant PP-seq labelled sequences that occurred during movement. Bottom: overall percentage breakdown of sequence occurrences during awake task execution (circles) and replayed during sleep (triangles). Dotted line is expected equal expression percentage. **f**. Percentage difference from equal expected replay expression for all recorded neural sequences. Data are coloured based on whether sequence expression was higher (boosted) or lower (supressed) than expected (n = 21 sessions, n = 8 mice). **g**. Schematic showing experimental design. Striatal recording sessions consisted of an initial period of sleep followed by task practice and then a second post-task sleep period. **h**. Mean replay rate per minute for pre and post task sleep recordings (Pairwise differences; two-sided one sample t-test; ***p < 0.001, Cohen’s d = 1.31). **i**. Mean replay event lengths (first to last spike duration. Pairwise differences; two-sided one sample t-test; *p = 0.030, Cohen’s d = 0.60). **j**. Relative individual neuron involvement frequencies for each sequence during awake task activity and sleep periods (MANOVA; Wilks lambda, *p = 0.018, η^2^ = 0.38) Shaded region = standard deviation. Panels h-j, n = 21 sessions, n = 8 mice. Boxplots show median, IQR and 1.5 IQR. Displayed stars: *p < 0.05, **p < 0.01, ***p < 0.001. Mouse drawings in **g** adapted from Scidraw under a Creative Commons license CC BY 4.0. Source data

To determine whether the neural sequences that we observed in the awake data were reactivated offline during sleep, we applied our awake trained PP-Seq model to post-task activity recorded during sleep (Fig. 3a) Neural sequences that were observed in the awake data were reactivated during sleep (Fig. 3b,c), but were not present in shuffled sleep data (circular shuffle, neuron IDs permuted; Extended Data Fig. 3a,b). Neural firing during replay followed a sequential ordering at the level of the single neurons (Fig. 3d and Extended Data Fig. 3c) and this ordering largely matched that observed for the same neurons during awake sequences (Fig. 3e and Extended Data Fig. 3d). At a macro-scale, neural sequences were often replayed individually but also occurred in combination (Fig. 3f,g). When combinations of neural sequences were replayed, they were more likely than chance to occur in the order that they appeared in the awake data as mice performed the behavioral sequence (Fig. 3h). This shows that both the sequential firing within a neural sequence and the compositional order between neural sequences were maintained during replay.

PP-Seq identifies neural sequences that occur across a range of temporal compressions; this feature revealed that striatal replay often progressed faster than real-world speed with the time course of the sequence being compressed (Fig. 3i,j). Although neural sequences were on average more likely to occur faster than they were in the awake recordings, a lot of the sequences also occurred at real-world speeds and could even progress slower, as is the case for hippocampal replay^33^. The individual reactivated neural sequences occurred in both forward and reverse directions. As has been detected in hippocampal post-task recordings^36^, a larger proportion of striatal replay occurred in the forward direction (Fig. 3j). Forward and reverse replay events occurred across the same range of speeds, with replay speeds ranging from >5× slower up to 20× faster than awake activity. Task-related neural sequences were more likely to be reactivated in post-task sleep compared to nontask-related sequences (Fig. 3k), suggesting that the task-related sequences were prioritized for reactivation. Individual task-related sequences were also differentially prioritized for replay (Extended Data Fig. 3e,f). In line with task-related sequences being prioritized, there was less replay during the pre-task sleep than the post-task sleep (Extended Data Fig. 3g–j). Like replay detected in the hippocampus, individual sequential events lasted around 100–400 ms (Fig. 3l)^12^. Also, neurons that were more frequently involved in replay events tended to be those that consistently participated in the same neural sequence during awake activity (Fig. 3m). This relationship was nonlinear such that the neurons that most consistently contributed to the awake neural sequence were more likely to occur in replay and the reverse was true for neurons that inconsistently contributed to the awake neural sequence. This suggests that, at the single-neuron level, cells that consistently contributed to the awake sequence were prioritized for replay. Together, these results show that both task-related neural sequences and individual neurons that were consistently active during the task are prioritized for replay.

**Extended Data Fig. 4:**
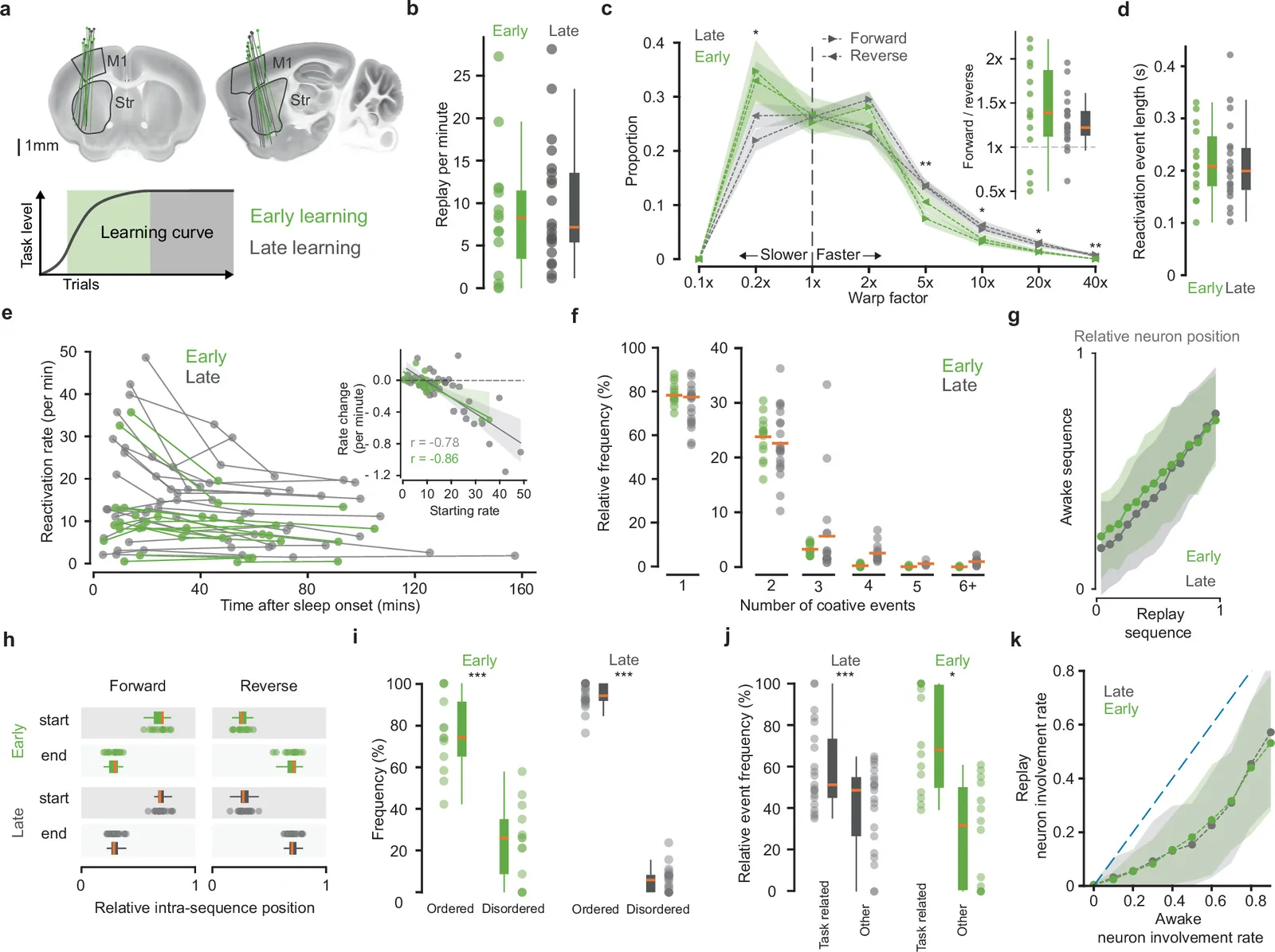
No difference was detected in procedural replay at different learning stages. **a**. Top: schematic showing implanted Neuropixel probe locations projected onto standard Allen atlas coronal (left) and sagittal (right). Bottom: schematic diagram showing early learning (before level 50) and late learning (after level 50) recording groups. **b**. Replay event rate per minute (two-sided independent t-test, p = 0.632, Cohen’s d = 0.16). **c**. Distribution of warp factors (1x represents awake speed) for forwards and backwards replay events (Early, forward vs reverse, PERMANOVA; p = 0.745, R^2^ = 0.60. Late, forward vs reverse, same as Fig. 4j. Early vs Late, PERMANOVA; **p = 0.004, R^2^ = 0.65. Pairwise PERMANOVA with Bonferroni correction; *p = 0.022, R^2^ = 0.46; **p = 0.007, R^2^ = 0.53; *p = 0.030, R^2^ = 0.56; *p = 0.011, R^2^ = 0.54; **p = 0.002, R^2^ = 0.36). Inset: proportion of forward vs reversed replays (1x represents equal proportion. Difference from chance, Early, two-sided one sample t-test; *p < 0.015, Cohen’s d = 0.75. Late; same as Fig. 4j inset. Early vs Late, two-sided independent t-test, p = 0.356, Cohen’s d = 0.32). **d**. Average (mean) single replay event lengths (duration from first to last spike) for each recording group (two-sided independent t-test, p = 0.869, Cohen’s d = 0.06). **e**. Main: reactivation rates for each analysed sleep epoch against time from first sleep onset. (Multivariate comparison between groups, MANOVA; Wilks lambda, p = 0.144, η^2^ = 0.07) Inset: rate change against starting rate for each pair of epochs per session. **f**. Frequency of single (isolated) and coactive events for each recording session (PERMANOVA, p = 0.082, R^2^ = 0.63). **g**. Mean relative positions in awake sequences vs. reverse replay sequences for all analysed neurons across every session (Shaded area = standard deviation. Multivariate comparison between groups, PERMANOVA; Wilks lambda, p = 0.833, R^2^ = 0.66). **h**. Average (mean) start and end points for all forward (top) and reverse (bottom) replay events. Position is relative to the corresponding average awake sequence (MANOVA; Wilks lambda, p = 0.682, η^2^ = 0.071). **i**. Relative frequencies of task ordered and disordered coactive sequences (Early: two-sided permutation test; ***p < 0.001, observed difference in means = 52.0%, 99th percentile permuted difference = 28.7, Cohen’s d = 2.81. Late: same as Fig. 4h. Difference for Early and Late: PERMANOVA; **p = 0.001, R^2^ = 0.40). **j**. Normalised percentages of task and non-task-related replay observed (Early: two-sided permutation test; ***p < 0.001, observed difference in means = 45.3%, 99th percentile permuted difference = 28.9%, Cohen’s d = 1.83. Late: same as Fig. 4k. Difference for Early and Late: PERMANOVA; p = 0.113, R^2^ = 0.57). **k**. Relative individual neuron involvement frequencies for each sequence during awake task activity and sleep periods (shaded area = standard deviation. Multivariate comparison between groups, MANOVA; Wilks lambda, p = 0.998, η^2^ = 0.0002). Panels b-k, Late: n = 21 sessions, n = 8 mice, Early: n = 15 sessions, n = 6 mice. Boxplots show median and interquartile range, shaded bands are SEM unless stated otherwise. Displayed stars: *p < 0.05, **p < 0.01, ***p < 0.001. Source data

**Extended Data Fig. 5:**
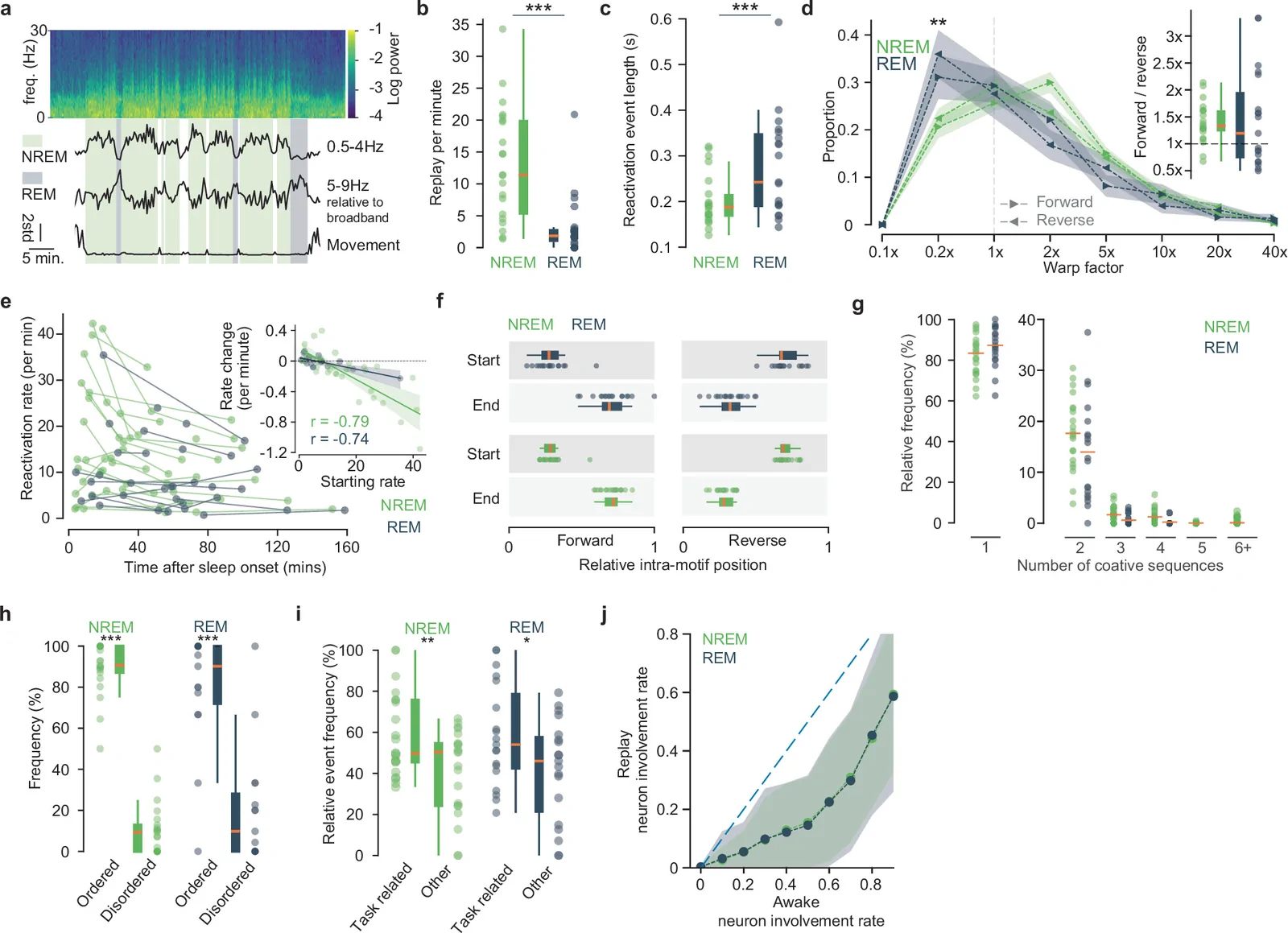
Replay occurs in both NREM and REM sleep. **a**. Spectrogram for an example sleep epoch with Delta and Theta band spectral power and average video tracking movement velocity underneath (traces). Sleep periods were NREM or REM periods classified from these metrics (see methods). **b**. Replay event rate per minute (two-sided Wilcoxon rank sum, ***p < 0.001, Rank-Biserial r = 0.88). **c**. Mean replay event lengths (first to last spike duration) for NREM and REM epochs (two-sided Wilcoxon rank sum, ***p < 0.001, Rank-Biserial r = 0.84). **d**. Main: distribution of warp factors (1x represents awake speed) for forwards and backwards replay events (forward vs reverse distribution: NREM, PERMANOVA; p = 0.139, R^2^ = 0.75. REM, PERMANOVA; p = 0.641, R^2^ = 0.73. NREM vs REM: PERMANOVA; ***p < 0.001, R^2^ = 0.69. Pairwise PERMANOVA with Bonferroni correction; **p = 0.004, R^2^ = 0.41). Shaded bands denote SEM. Inset: proportion of forward vs reversed replays (1x represents equal proportion), (Difference from chance: NREM, two-sided one sample t-test; ***p < 0.001, Cohen’s d = 1.06, REM, two-sided one sample t-test, *p < 0.041, Cohen’s d = 0.50. NREM vs REM; two-sided Wilcoxon rank sum; p = 0.984, Rank-Biserial r = 0.01). **e**. Main: reactivation rates for each analysed sleep epoch against time from first sleep onset. (Multivariate comparison between groups, MANOVA, Wilks lambda, p = 0.149, η^2^ = 0.08). Inset: rate change against starting rate for each pair of epochs per session (NREM; OLS; r = -0.79, R^2^ = 0.63, ***p < 0.001. REM; OLS; r = -0.74, R^2^ = 0.55, *p = 0.002). **f**. Mean start and end points for all forward (left) and reverse (right) NREM and REM replay events. Position is relative to the corresponding average awake sequence (PERMANOVA; Wilks lambda, p = 0.768, R^2^ = 0.75). **g**. Frequency of single (isolated) and concatenated events for each recording session (PERMANOVA; p = 0.139, R^2^ = 0.66). **h**. Relative frequencies of task ordered and disordered concatenated sequences with respect to observed awake task ordering for recordings containing 4 or more task related sequences (see methods). NREM, two-sided permutation test; ***p < 0.001, observed difference in means = 78.4%, 99th percentile permuted difference = 30%, Cohen’s d = 6.03. REM, two-sided permutation test; ***p < 0.0001, observed difference in means = 58.6%, 99th percentile permuted difference = 34.6%, Cohen’s d = 2.03. Difference for NREM and REM, PERMANOVA; p = 0.167, R^2^ = 0.45). **i**. Normalised percentages of task and non-task related replay observed (NREM, two-sided permutation test; **p = 0.003, observed difference in means = 18.5%, 99th percentile permuted difference = 16.1%, Cohen’s d = 0.89. REM, two-sided permutation test; *p = 0.022, observed difference in means = 16.9%, 99th percentile permuted difference = 19.4%, Cohen’s d = 0.89. Multivariate comparison between groups, PERMANOVA; p = 0.901, R^2^ = 0.63). **j**. Relative individual neuron involvement frequencies for each sequence during awake task activity and sleep periods (Multivariate comparison between groups, MANOVA; Wilks lambda, p = 0.998, η^2^ = 0.0002). Shaded region = standard deviation. n = 21 sessions, n = 8 mice. Boxplots show median, IQR and 1.5 IQR. Displayed stars: *p < 0.05, **p < 0.01, ***p < 0.001. Source data

Analysis of the composition of each reactivated neural sequence revealed that replay tended to preferentially involve neurons that made up the central portion, rather than the boundaries, of each task-related neural sequence (Fig. 3n). The rate of replay also tended to decay from sleep onset and there was a significant relationship between time from first sleep onset and replay rate (Fig. 3o). Analyzing the decay rate compared to the current replay rate, we found a strong relationship suggesting that replay rate had nonlinear decay toward an equilibrium rate. Finally, analysis of recordings done during early learning revealed that there was a larger proportion of time-compressed replay events in sessions from expert mice; otherwise we were unable to detect any differences in the replay characteristics between early and late stages of learning (Extended Data Fig. 4). Striatal replay also occurred during both slow-wave nonrapid eye movement (NREM) and rapid eye movement (REM) sleep (Extended Data Fig. 5). The rate of replay during REM sleep was lower than during NREM sleep and there was a larger proportion of replay that occurred at slower than real-world speeds during REM sleep (Extended Data Fig. 5b,c). We could not detect any difference between the other features of replay across the two sleep states.

**Extended Data Fig. 6:**
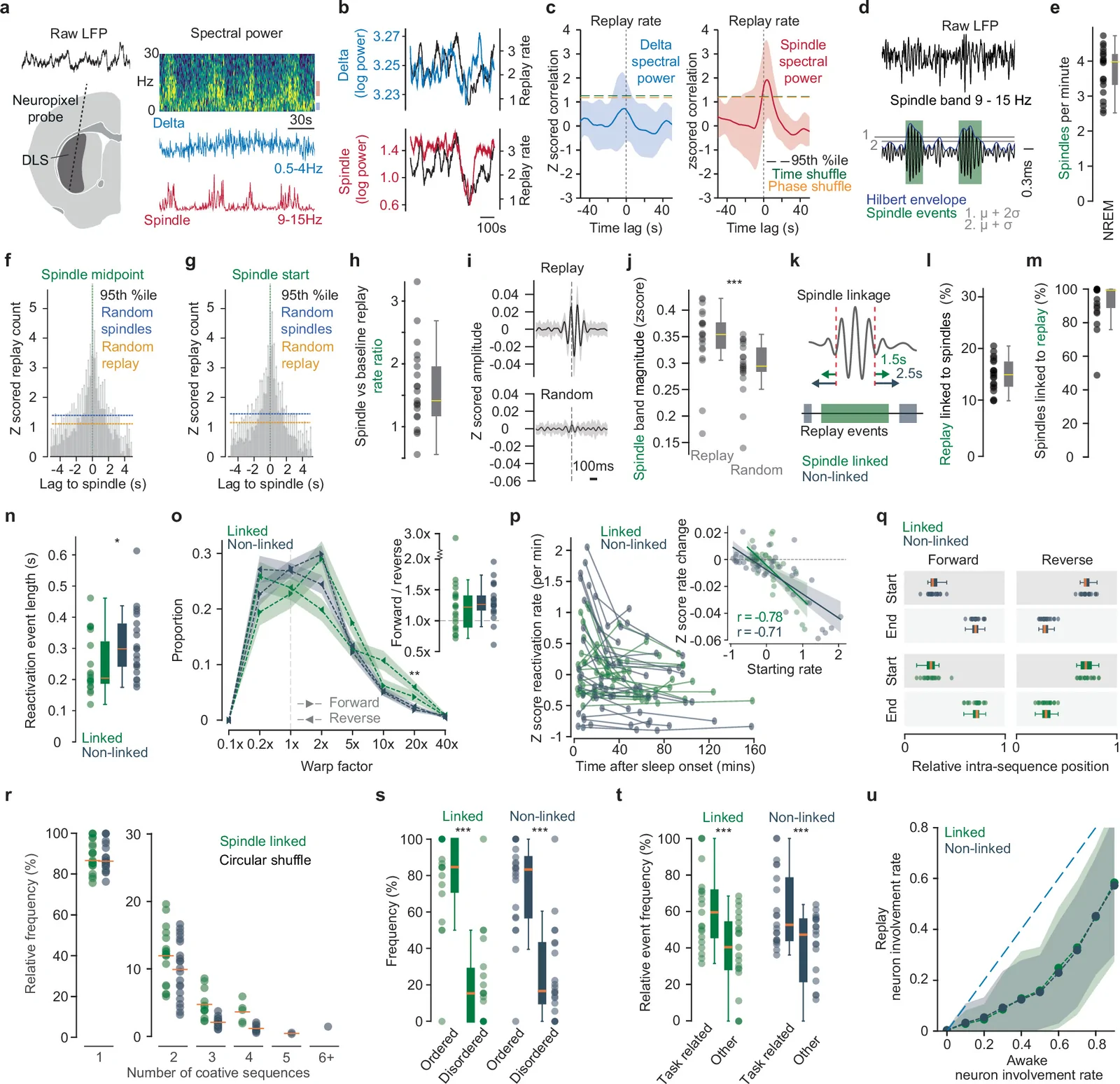
Relationship between replay and sleep spindles. **a**. Left: schematic of Neuropixel probe location and example raw LFP trace. Right: spectrogram for example sleep epoch with corresponding extracted spindle and delta spectral power traces. **b**. Example epoch showing smoothed delta (top) and spindle (bottom) log spectral power against smoothed binned replay rate (1 s increment bins, 30 bin smoothing window). **c**. Left: Cross-correlation between smoothed log delta band power and replay rate. Right: Cross-correlation between smoothed spindle-band power and replay rate. Each plot shows Pearson correlation coefficient as a function of time lag. Dotted horizontal lines represent the 95th percentile of shuffled data from randomly permuting spectral power time bins (green) or Fourier transformed phase angles (yellow, see methods). **d**. Schematic showing an example raw LFP trace and the corresponding signal after band pass at spindle frequency. Spindle events were identified by doubly thresholding the Hilbert envelope of this signal. **e**. Spindle rate per minute. **f**. Binned distance to closest spindle midpoint for each replay. Dotted lines represent the 95th percentile for random data for randomly selected replay (orange) and spindle (**blue**) times. **g**. Binned distance to closest spindle start for each replay. Dotted lines represent the 95th percentile for random data for randomly selected replay (orange) and spindle (**blue**) times. **h**. Ratio of replay occurring inside spindle events to replay outside of spindles. Normalised by time in each state. **i**, Event triggered average for replay and random events. **j**. Mean spindle band magnitude (area under Hilbert envelope) during replay events and for random time periods (two-sided Wilcoxon rank sum; ***p < 0.001, Rank-Biserial r = 1.0). **k**. Schematic showing replay linkage classification. **l**. Percentage of all repay events that were linked (within 1.5 s) of a spindle. **m**. Percentage of all sleep spindles that were linked (within 1.5 s) of replay. **n**. Mean replay event lengths (first to last spike duration) for spindle linked and non-linked events (two-sided Wilcoxon rank sum; *p < 0.024, rank, Biserial r = 0.57). **o**. Main: distribution of warp factors (1x represents awake speed) for forwards and backwards replay events (Linked, forward vs reverse: PERMANOVA; *p = 0.011, R^2^ = 0.75. Unlinked, forward vs reverse: PERMANOVA; p = 0.097, R^2^ = 0.76. Linked vs unlinked: PERMANOVA; *p = 0.012, R^2^ = 0.76; pairwise PERMANOVA with Bonferroni correction; **p = 0.004, R^2^ = 0.52). Inset: proportion of forward vs reversed replays (1x represents equal proportion), (Difference from chance: Linked, one sample t-test; *p < 0.032, Cohen’s d = 0.50, Unlinked, two-sided one sample t-test, ***p < 0.001, Cohen’s d = 1.06. Linked vs Unlinked; two-sided Wilcoxon rank sum; p = 0.168, Rank-Biserial r = 0.35). **p**. Main: reactivation rates for each analysed sleep epoch against time from first sleep onset. (Multivariate comparison between groups, MANOVA; Wilks lambda, p = 0.481, η^2^ = 0.02). Inset: rate change against starting rate for each pair of epochs per session (linked; OLS; r = -0.78, R^2^ = 0.61, ***p < 0.001. unlinked; OLS; r = -0.71, r^2^ = 0.51, ***p < 0.001). **q**. Mean start and end points for all forward (left) and reverse (right) spindle linked and unlinked replay events. Position is relative to the corresponding average awake sequence (MANOVA; Wilks lambda, p = 0.825, η^2^ = 0.04). **r**. Frequency of single (isolated) and concatenated events for each recording session for spindle linked replay vs replay linked to event times drawn from circular shuffle of the spindle times (see methods. PERMANOVA; p = 0.629, R^2^ = 0.71). **s**. Relative frequencies of task ordered and disordered concatenated sequences with respect to observed awake task ordering for recordings containing 4 or more task related sequences (see methods). Linked, two-sided permutation test; ***p < 0.001, observed difference in means = 56.5%, 99th percentile permuted difference = 29.5%, Cohen’s d = 2.17. Unlinked, permutation test; ***p < 0.001, observed difference in means = 49%, 99th percentile permuted difference = 25.2%, Cohen’s d = 1.95. Difference for NREM and REM, PERMANOVA; p = 0.636, R^2^ = 0.52). **t**. Normalised percentages of task and non-task related replay observed (Linked, two-sided permutation test; ***p < 0.001, observed difference in means = 23.8%, 99th percentile permuted difference = 17.3%, Cohen’s d = 1.10. Unlinked, two-sided permutation test; ***p < 0.001, observed difference in means = 21.8%, 99th percentile permuted difference = 16.4%, Cohen’s d = 1.06. Multivariate comparison between groups, MANOVA; Wilks lambda, p = 0.876, R^2^ = 0.62. **u**. Relative individual neuron involvement frequencies for each sequence during awake task activity and sleep periods (Multivariate comparison between groups, MANOVA; Wilks lambda, p = 0.995, η^2^ = 0.0007). n = 21 sessions, n = 8 mice. Boxplots show median IQR and 1.5 IQR. Displayed stars: *p < 0.05, **p < 0.01, ***p < 0.001. Source data

In other structures, replay has been linked with the occurrence of high-frequency oscillations such as SW-R in the hippocampus^34^ or sleep spindles in the cortex^37,38^. In the striatum, sleep spindles are thought to be critical for offline motor consolidation because they lead to an increase in corticostriatal communication that facilitates plasticity^28^. In line with a link between spindles and replay there was a positive correlation between replay rate and spindle band spectral power and the rate of replay peaked around the midpoint of detected sleep spindles (Extended Data Fig. 6a–g). In line with this, there was an increased rate of replay during sleep spindles (Extended Data Fig. 6h). Although this points to an important modulatory role of spindles on the rate of replay, only a small fraction of replay was temporally linked to the occurrence of a sleep spindle (Extended Data Fig. 6k,l). In contrast, we could detect replay in almost every sleep spindle (Extended Data Fig. 6m). Spindle-linked replay events tended to be shorter and were more likely to be time compressed than nonlinked replay events (Extended Data Fig. 6n,o). We could not detect any differences in the other characteristics of replay between spindle-linked and nonlinked events (Extended Data Fig. 6p–u). Together this suggests that sleep spindles are not a necessary driver of striatal replay, but that these oscillations do play a critical role in modulating the rate and occurrence of striatal replay.

**Extended Data Fig. 7:**
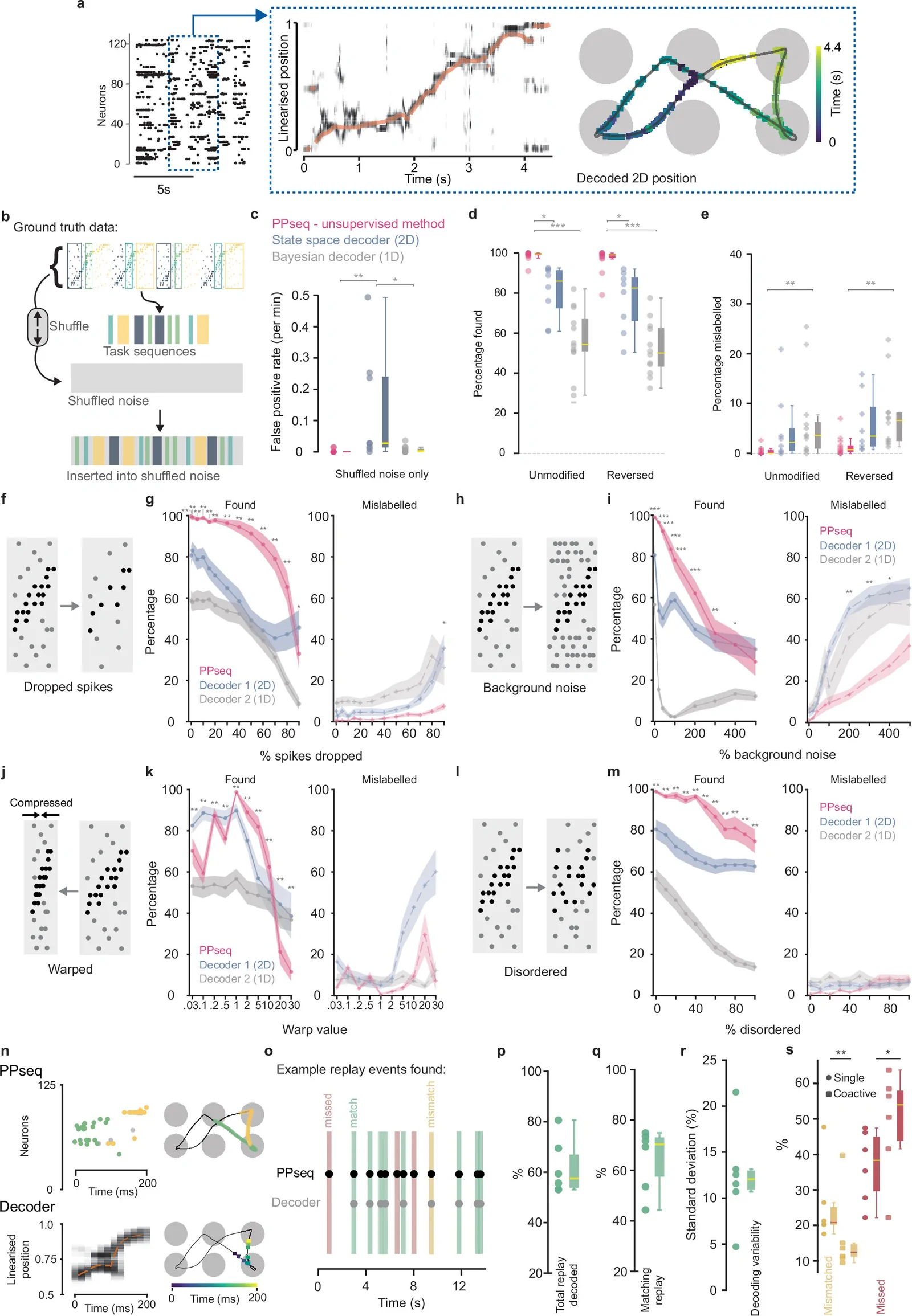
Unsupervised replay detection outperforms a state-of-the-art decoding approach on ground truth data. **a**. Left: raster plot showing striatal neural activity during task execution. Blue highlighted region is an example of spiking during a single task trial. Middle: Linearised trajectory of the task. Shading shows the posterior likelihood (white = 0, darker = more likely) across all linearised spatial bins (average in orange) Right: decoded 2D trajectory of a single trial activity, each square corresponds to the weighted average posterior probability across 2D space projected to an average trajectory of the task. **b**. Schematic showing production of ground truth ‘synthetic replay’. Time periods containing PP-seq identified neural sequences were extracted and pseudo-randomly implanted into spike matched shuffled data (neuron ID’s permuted). **c**. False positive rates (events detected per minute in shuffled background noise) for PP-Seq and the decoders (Kruskal-Wallis; **p = 0.007, η^2^ = 0.30. post-hoc Dunn test; **p = 0.006, Rank-Biserial r = 0.53. *p = 0.049, Rank-Biserial r = 0.41). **d**. Percentage of normal (unmodified) and reverse implanted sequences that were correctly identified by PP-Seq and the decoders (Unmodified: Kruskal-Wallis; ***p < 0.001, η^2^ = 0.30. Post-hoc Dunn test; *p = 0.028, Rank-Biserial r = 0.94. ***p < 0.001, Rank-Biserial r = 0.77. Reversed: Kruskal-Wallis; ***p < 0.001, η^2^ = 0.74. Post-hoc Dunn test; *p = 0.042, Rank-Biserial r = 0.88. ***p < 0.001, Rank-Biserial r = 1.0). **e**. Percentage of normal and reverse implanted sequences that were identified but mislabelled as the wrong sequence type by PP-Seq and the decoders (Unmodified: Kruskal-Wallis; **p = 0.003, η^2^ = 0.33. post-hoc Dunn test; **p = 0.002, Rank-Biserial r = 0.76. Reversed: Kruskal-Wallis; **p = 0.001, η^2^ = 0.37. post-hoc Dunn test; **p = 0.001, Rank-Biserial r = 0.81). **f**. Schematic diagram of test done: each implanted sequence period is modified to have a percentage of spikes removed. **g**. Left: For different percentage spike drop out, the mean percentage of sequence that were correctly identified by PP-Seq and the two decoder methods (PERMANOVA; ***p < 0.001, R^2^ = 0.06. Post hoc: feature-wise PERMANOVA with Bonferroni correction). Right: mean percentage of implanted sequence periods that were identified but mislabelled as the wrong sequence type by PP-Seq and the decoders (PERMANOVA; **p = 0.001, R^2^ = 0.05. Post hoc: feature-wise PERMANOVA with Bonferroni correction). **h**. Schematic diagram of test done: each implanted sequence period is modified to have a percentage of background noise randomly added. **i**. Left: For different percentage background noise insertion, the mean percentage of sequences that were correctly identified by PP-Seq and the two decoder methods (PERMANOVA; ***p < 0.001, R^2^ = 0.07. Post hoc: feature-wise PERMANOVA with Bonferroni correction). Right: mean percentage of implanted sequence periods that were identified but mislabelled as the wrong sequence type by PP-Seq and the decoders (PERMANOVA; ***p < 0.001, R^2^ = 0.07. Post hoc: feature-wise PERMANOVA with Bonferroni correction). **j**. Schematic diagram of test done: each implanted sequence period is stretched or compressed by a warp factor. **k**. Left: For different warp values, the mean percentage of sequences that were correctly identified by PP-Seq and the two decoder methods (PERMANOVA; ***p = 0.001, R^2^ = 0.06. Post hoc: feature-wise PERMANOVA with Bonferroni correction). Right: mean percentage of implanted sequence periods that were identified but mislabelled as the wrong sequence type by PP-Seq and the decoders (PERMANOVA; p = 0.051, R^2^ = 0.03. Post hoc: feature-wise PERMANOVA with Bonferroni correction). **l**. Schematic diagram of test done: each implanted sequence period is discorded by randomly swapping a percentage of spikes. **m**. Left: For different percentage of the spikes disordered, the mean percentage of sequences that were correctly identified by PP-Seq and the two decoder methods (PERMANOVA; ***p < 0.001, R^2^ = 0.06. Post hoc: feature-wise PERMANOVA with Bonferroni correction). Right: mean percentage of implanted sequence periods that were identified but mislabelled as the wrong sequence type by PP-Seq and the decoders (PERMANOVA; p = 0.051, R^2^ = 0.03). For panels c-m, for each test value and for each group (PP-Seq and Decoders), n = 10 sessions from n = 6 implanted animals. **n**. Top: PP-Seq labelled spikes (left) and corresponding PP-seq sequence positions observed during task activity projected onto average tracking trajectory (right). Bottom: (left) for the spikes shown above, the decoded 1D position across linearised task space (red dashed line). Shading shows the posterior likelihood across all spatial bins (white = 0, darker = more likely). Maximum of the 1D decoded position (the most probable position) projected onto 2D average tracking trajectory (right). **o**. Example epoch showing PP-Seq identified replay events and replays that were also found by the decoder. Perfect matches (green markers), replays with mismatched spatial locations (yellow) and PP-seq replay not found by the decoder (red) are shown. **p**. Percentage of PP-Seq replay events that were also found by the decoder. **q**. The percentage of found events which were spatially harmonious between the two methods. **r**. Standard deviation of total found percentages between sequence types for each session. **s**. Percentage mismatched and missed sequences for decoded epochs which contained either a single PP-Seq sequence (circle) or multiple coactive sequences (square) (two-sided paired t-test with Bonferroni correction, Cohen’s d; **p = 0.001, d = 0.74 *p = 0.010, d = 0.62,). n = 6 sessions, n = 6 mice, for panels p-s. Boxplots show median IQR and 1.5 IQR, shaded bands = SEM. Displayed stars: *p < 0.05, **p < 0.01, ***p < 0.001. Source data

To benchmark our unsupervised method for replay detection, we compared it with two Bayesian decoding methods, a classic linear decoding method^39^ and a state-of-the-art combined state–space and nonlinear decoding method^33^. On synthetic data, designed to create a ground-truth dataset, PP-Seq was both more accurate and more sensitive at detecting replay than either of the decoding methods (Extended Data Fig. 7a–e). PP-Seq was also more robust at finding replay when noise, jitter and temporal distortions were applied to the data (Extended Data Fig. 7f–m). In our actual recording data, most PP-Seq-identified replay events were also identified by the nonlinear decoding method, validating that multiple methods identify replay of our task-related activity in the striatum during sleep (Extended Data Fig. 7n–s).

In summary, applying PP-Seq to post-task sleep revealed that neural sequences that occurred during the awake experience were replayed during NREM and REM sleep. The features of this replay were consistent with replay in other areas such as the hippocampus, in that it occurred at time-compressed and real-world speeds and occurred in both forward and reverse orders. The striatal replay appeared to be structured in a compositional manner such that individual neural sequences could be replayed individually, in awake order or even in combinations that rarely occurred during behavior. The replay was also prioritized at the level of both the type of neural sequence and the individual neurons within a sequence, consistent with the idea that replay is important for the consolidation of both sequential order and refinement of the execution of individual parts of the behavioral sequence.

### Bidirectional relationship between behavior and replay

**Fig. 4:**
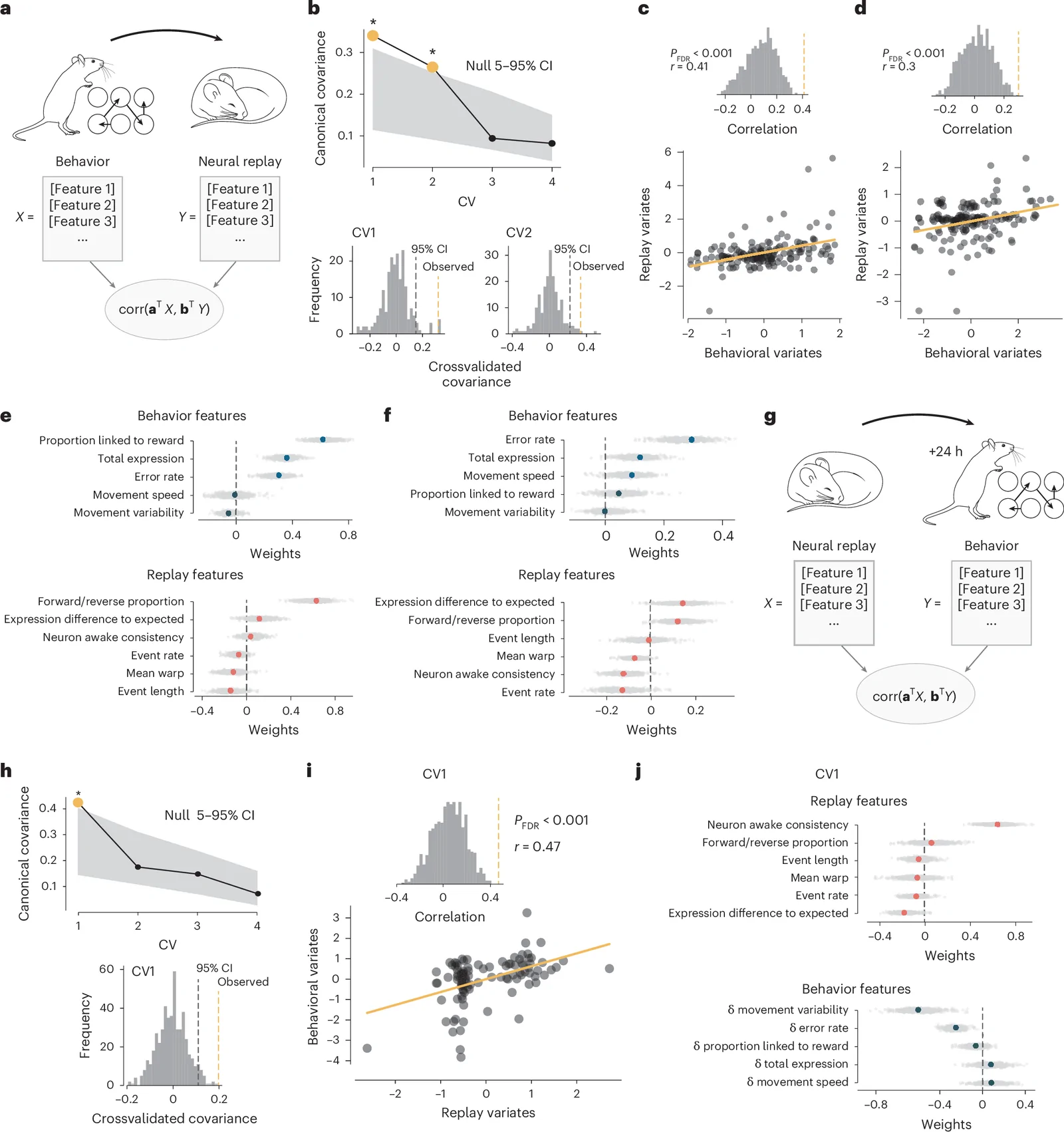
Bidirectional relationship between behavior and replay. **a**, Schematic diagram showing that the CCA determines whether features of behavior correlated with features of post-task neural replay. **b**, Top: magnitude of the canonical covariation between behavioral and replay features explained by each CV. The overall multivariate model testing the relationship between features of behavior and features of post-task neural replay was statistically significant (Wilks’ *λ* = 0.712, *F*(30, 582) = 1.73, *P* < 0.001). The 5th to 95th percentiles of the null distribution of the same measures, estimated via a permutation test (Methods), are shown in gray. Bottom: for the CVs that passed the initial permutation test, mean canonical covariance observed from fivefold crossvalidated data (yellow line) is shown against the distribution of fold-wise permuted data (the 95th percentile of permuted distribution is the black line). CVs that passed both testing paradigms are highlighted in yellow (top). **c**, Scatter plot of behavioral and replay variates (linear combinations of multivariate features) for CV1. Inset: distribution of CCA correlations from permutation testing (the yellow dash representing the actual correlation). **d**, Same as **c** but for CV2. **e**, Colored dots representing individual behavioral and replay feature weights for CV1 (*n* = 35 sessions, *n* = 10 mice). Gray dots are weights from bootstrapped resampling (see Methods for details). **f**, Same as **e** but for CV2. **g**, Schematic diagram showing that the CCA determines whether features of replay correlated with changes in behavioral performance on the subsequent day. **h**–**j**, Same as **b**–**f** but for the first CV of the CCA described in **g** (*n* = 23 sessions, *n* = 10 mice). The overall multivariate model testing the relationship between features of replay and changes in behavioral performance was statistically significant (Wilks’ *λ* = 0.681, *F*(30, 362) = 1.21, *P* = 0.02). ^*^P < 0.05. corr, correlation; FDR, false recovery rate. Mice drawings in **a** and **g** adapted from Scidraw under a Creative Commons license CC BY 4.0. Source data

**Extended Data Fig. 8:**
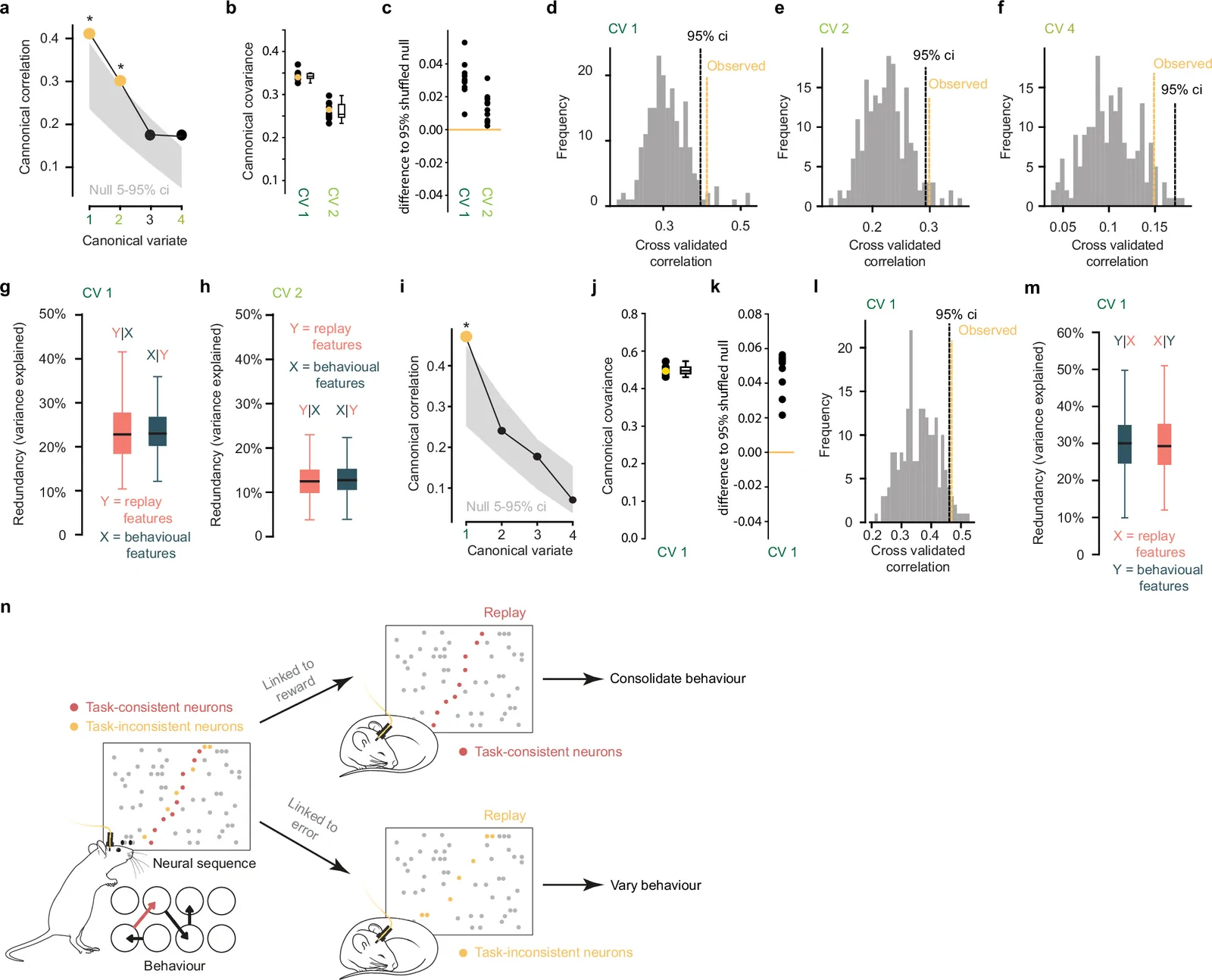
CCA for the link between behaviour and replay. **a**. Linear canonical correlation strength between behaviour and replay variables plotted for the first four canonical variates. The 5th to 95th percentiles of the null distribution, estimated via permutation testing, are shown in grey. Canonical variates which passed both testing paradigms are highlighted yellow. **b**. Magnitude of the canonical covariation between behavioural and replay features explained by the top two canonical variate after excluding one subject at a time. (n = 10 mice) **c**. The difference between the canonical covariation and the 95th percentiles of the null distribution in each case where a subject is excluded (n = 10 mice). **d**. For the canonical variates (CV) which passed the initial permutation test, mean canonical covariance observed from 5-fold cross validated data (yellow line) is shown against the distribution of fold-wise permuted data (95th percentile of permuted distribution is the black line) for CV1. **e**. Same as panel d but for CV2. **f**. Same as panel d but for CV4. **g.h**. Directional variances explained (redundancy) by canonical variate 1 (panel **g**) and 2 (panel **h**) (n = 35 sessions, n = 10 mice) **i**. Linear canonical correlation strength between replay features and the change in behavioural metrics the subsequent day (n = 23 sessions, n = 10 mice). **j**. Magnitude of the canonical covariation between replay features and the change in behavioural metrics the subsequent day explained by the top canonical variate after excluding one subject at a time (n = 10 mice). **k**. The difference between the canonical covariation and the 95th percentiles of the null distribution in each case where a subject is excluded (n = 10 mice). **l**. For the canonical variate (CV1) which passed the initial permutation test, mean canonical covariance observed from 5-fold cross validated data (yellow line) is shown against the distribution of fold-wise permuted data (95th percentile of permuted distribution is the black line). **m**. Directional variances explained (redundancy) by canonical variate 1 (n = 10 mice). **n**. Schematic summary of the CCA results. Boxplots show median IQR and 1.5 IQR, Displayed stars: *p < 0.05, **p < 0.01, ***p < 0.001. Source data

To determine the relationships between behavior and post-task replay, we used canonical correlation analysis (CCA) (Fig. 4a), an unsupervised learning technique that seeks to find correlations between two multidimensional datasets. The CCA identified specific patterns (‘canonical variates’) of behavior that were linked to distinct combinations of replay features. Based on their covariance explained, we selected the first two canonical variates (CVs) for further analysis (Fig. 4b–d and Extended Data Fig. 8). Each CV represented a distinct pattern that relates a weighted set of behavioral metrics to a weighted set of replay features. In the first dimension, the most heavily weighted feature of behavior was the proportion of times that a neural sequence was followed by a reward; this was linked positively to how often that neural sequence was replayed in the forward direction, how often it would be replayed relative to the other sequences and whether replay events were composed of neurons that consistently participated during the awake behavior (Fig. 4e). In the second dimension, the most heavily weighted feature of behavior was the error rate associated with a particular neural sequence; this was also linked to how often the sequence was replayed in the forward direction and how much it would preferentially be replayed (Fig. 4f). However, this pattern of behavioral features was linked to negative weighting for whether the replay of a neural sequence would contain neurons that consistently participated in the same neural sequence during awake activity (Fig. 4f). This suggests that neural sequences that were associated with salient behavioral events were prioritized for replay and replayed more often in the forward direction. However, replay of sequences that are linked to reward are more likely to be composed of neurons that consistently participated in the awake neural sequence, whereas the opposite is true for sequences that were associated with errors (Extended Data Fig. 8).

To determine how the content of replay influenced changes in task performance on the next day, we performed a CCA that assessed the link between features of replay and changes in subsequent task performance (Fig. 4g). Based on their covariance explained, we selected the first CV (CV1) for further analysis (Fig. 4h,i and Extended Data Fig. 8i–m). In this dimension, the most heavily weighted feature of replay was how consistently neurons in the replay sequence had participated in the awake neural sequence; there was also a positive weighting on the proportion of times that the neural sequence was played in the forward direction (Fig. 4j). These features of replay were most heavily linked to a decrease in the movement variability associated with that neural sequence, as well as a decrease in error rate associated with that portion of the behavioral sequence and an increase in speed. It is interesting that the rate of replay or how much an individual neural sequence was prioritized was not positively linked to changes in next-day behavior. This suggests that it is the content and not the quantity of replay that is linked to changes in task performance on the subsequent day.

Taken together, neural sequences that are linked to behaviorally salient events are prioritized for replay and are more likely to be replayed in a forward direction. When neural sequences are linked to rewards, they are more likely to contain neurons that consistently participated in the awake sequence and the opposite is true when the neural sequences are linked to errors. In turn, we found that the degree to which replay events contain neurons that were consistently task active during the awake behavior, and whether replay is in the forward direction, impact the degree by which replay events are linked to changes in task performance (Extended Data Fig. 8n).

### Procedural memory formation and replay are independent of the hippocampus

**Fig. 5:**
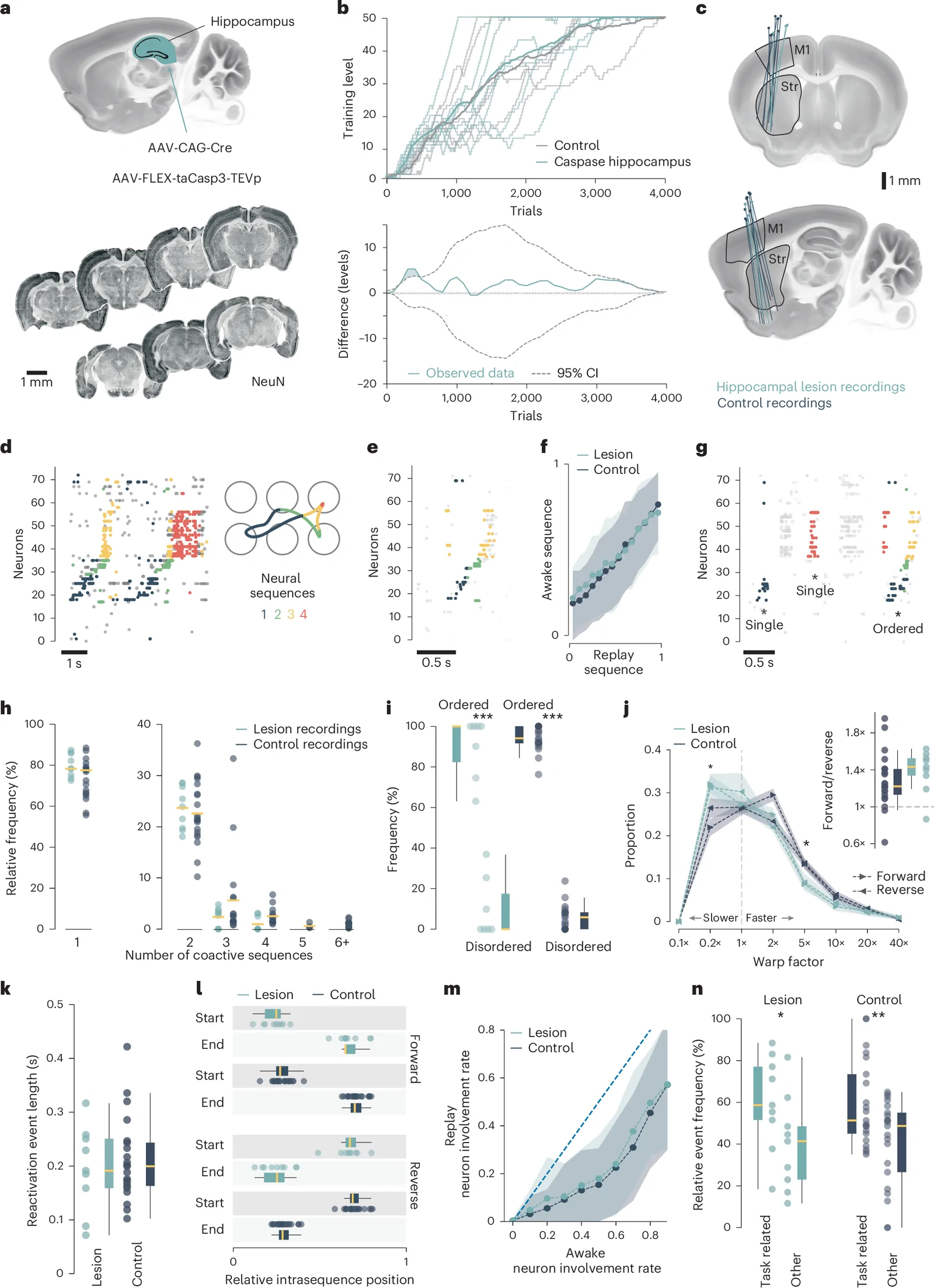
Procedural memory formation and replay are independent of the hippocampus. **a**, Top: schematic of the experimental approach for bilateral lesion of the hippocampus. Bottom: histology, example NeuN-stained coronal slices showing lesion extent across hippocampal volume for a mouse. **b**, Top: learning progression curves (training level versus trials) for control and lesioned animal groups (shaded area = s.d.). Bottom: differences in performance between the groups. The dotted lines indicate the 95% CI for the shuffled data (Methods; lesion *n* = 7 mice, control *n* = 6 mice). **c**, Schematic showing implanted Neuropixel probe locations projected onto a standard Allen coronal (top) and sagittal (bottom) atlas. **d**, PP-Seq-labeled sequences for example task related to awake spikes. **e**, Example replay (same neurons as in **d**) of concatenated sequences that are ordered with respect to task-related activity. **f**, Mean relative spiking positions in awake sequences versus replay sequences for all analyzed neurons across every session for lesion and control animals (shaded area = s.d.). Multivariate comparison between groups is with PERMANOVA: *P* = 0.922, *R*^2^ = 0.66. **g**, Example sleep period with PP-Seq-labeled replay sequences. **h**, Frequency of single (isolated) and concatenated events for each recording session (PERMANOVA; *P* = 0.136, *R*^2^ = 0.62). **i**, Relative frequencies of task-ordered and task-disordered concatenated sequences with respect to observed awake task ordering for recordings containing four or more task-related sequences (Methods) (lesion, two-sided permutation test, ^***^P = 0.038, observed difference in means = 79.4%, 99th percentile permuted difference = 53.4%, Cohen’s *d* = 5.27; control, same as Fig. 4h; difference for lesion and control, PERMANOVA, *P* = 0.255, *R*^2^ = 0.49). **j**, Distribution of warp factors (1× represents awake speed) for forward and backward replay events (lesion, forward versus reverse, PERMANOVA, *P* = 0.744, *R*^2^ = 0.71; control, forward versus reverse, same as Fig. 4j; lesion versus control, PERMANOVA, ^**^P = 0.004, *R*^2^ = 0.72; pairwise PERMANOVA with Bonferroni’s correction, ^*^P = 0.042, *R*^2^ = 0.51; ^*^P = 0.014, *R*^2^ = 0.53). Inset: proportion of forward versus reversed replays (1× represents equal proportion; difference from chance, lesion, two-sided, one-sample Student’s *t*-test, ^**^P = 0.001, Cohen’s *d* = 1.64; control, same as Fig. 4j). Inset: lesion versus control, two-sided, independent Student’s *t*-test, *P* = 0.359, Cohen’s *d* = 0.37. **k**, Average (mean) replay event lengths observed for each recording session (two-sided, independent Student’s *t*-test, *P* = 0.59, Cohen’s *d* = 0.22). **l**, Mean start and end points for all forward (top) and reverse (bottom) replay events. The position is relative to the corresponding average awake sequence (multivariate ANOVA (MANOVA): Wilks’ *λ*, *P* = 0.426, *η*^2^ = 0.14). **m**, Relative individual neuron involvement frequencies for each sequence during awake task activity and sleep periods (multivariate comparison between groups, MANOVA: Wilks’ *λ*, *P* = 0.668, *η*^2^ = 0.05; shaded region = s.d.). **n**, Normalized percentages of task-related and nontask-related replay observed (lesion, two-sided permutation test; *P* = 0.038, observed difference in means = 20.3.9%, 99th percentile permuted difference = 26.0%; control, same as Fig. 4k; multivariate comparison between groups, MANOVA; Wilks’ *λ*, *P* = 0.996, *η*^2^ = 0.0003). In **f** and **h**–**n**: control, *n* = 21 sessions, *n* = 8 mice; lesion, *n* = 9 sessions, *n* = 3 mice. The boxplots show the median, IQR and range up to 1.5× the IQR. Shaded bands denote s.e.m. unless stated otherwise. ^*^P < 0.05, ^**^P < 0.01, ^***^P < 0.001. Source data

**Extended Data Fig. 9:**
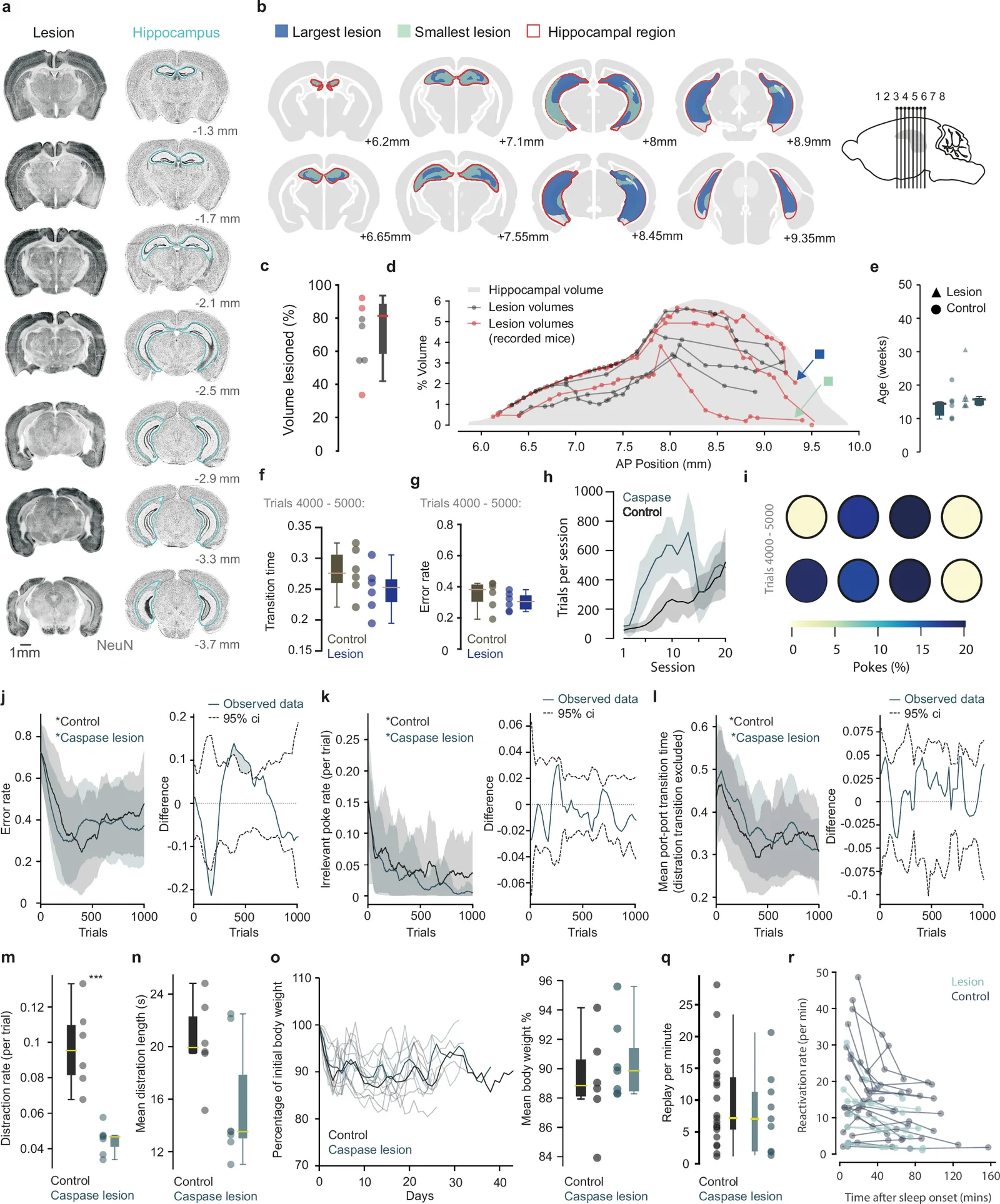
Histology and quantification for hippocampal lesions. **a**. Left: histology for example NeuN stained coronal slices showing lesion extent across hippocampal volume. Right: corresponding Allen reference atlas slices with hippocampal volume highlighted (blue outline). **b**. Largest (blue shaded) and smallest (green shaded) lesion extent for coronal slices across the extent of the hippocampus. **c**. Percentage of total hippocampal volume lesioned for each animal. Mice that were used in Neuropixel recording experiments are shown in red. **d**. Percentage of total hippocampal volume lesioned for each animal across the extent of the hippocampus. Total hippocampal volume is shown (grey). Smallest and largest lesions are labelled by blue and green markers respectively. Mice that were used in Neuropixel recording experiments are shown in red (n = 7 mice). **e**. Ages for all control and lesioned mice (Wilcoxon rank-sum; p = 0.279, Rank-Biserial r = 0.38). **f**. Average (mean) transition port-to-port transition times for expert mice in each group. (two-sided independent t-test; p = 0.225, Cohen’s d = 0.75). **g**. Port-to-port transition errors for expert (trials 4000-5000) mice in lesion and control groups. (two-sided independent t-test; p = 0.364, Cohen’s d = 0.55). **h**. Mean number of trials performed per session over the course of 20 training days for control and lesion group mice (shaded area is standard deviation). **i**. Average (mean) percentage port poke occurrences for mice in the lesion group (+/-SEM shown in grey). **j**. Left: mean port-port error rate evolution across first 1000 trials (shaded area is standard deviation). Right: differences in error rate between observed lesion data and group shuffle. Dotted lines indicate the 95% confidence interval for the shuffled data (see methods). **k**. Left: poke rate into non-task-related ports across the first 1000 trials. Right: differences in irrelevant poke rate between observed lesion data and group shuffle (as in panel j). **l**. Left: mean port-port transition times for control and hippocampus lesion animals across the first 1000 trials. Right: differences in port-port transition times between observed lesion data and group shuffle (as in panel j). **m**. Distraction rate (frequency port-port transitions exceeding 2 s) for the first 1000 trial epoch (two-sided independent t-test; ***p = 0.000185). **n**. Mean distraction event length over first 1000 trial epoch (two-sided independent t-test; ***p = 0.0594). **o**. Body weight percentage per mouse across training duration. **p**. Mean body weight percentage over training epoch (two-sided independent t-test; p = 0.441). Panels e – p; Control n = 6 mice, Lesion n = 6 mice **q**. Replay event rate per minute for lesion and control time epochs (two-sided independent t-test p = 0.527, Cohen’s d = 0.255). **r**. Reactivation rates for each analysed sleep epoch against time from first sleep onset (Multivariate comparison between groups, p = 0.0897, MANOVA, η^2^ = 0.0854). Panels q,r; Control: n = 21 sessions, n = 8 mice. Lesion: n = 9 sessions, n = 3 mice.Boxplots show median IQR and 1.5 IQR, Displayed stars: *p < 0.05, **p < 0.01, ***p < 0.001. Source data

Having established that task-related neural activity is reactivated offline and shown that it shares many features with previously observed hippocampal replay, we next aimed to investigate whether these similarities were indicative of shared, mechanistic dependency. Indeed, even for ultimately hippocampus-independent memories, initial consolidation is thought to be organized by hippocampal dynamics^1,2,6–8,11^. Hippocampal SW-R events have been shown to display consolidation-dependent coupling with replay in cortical areas^11,34,40–42^, suggesting that hippocampal dynamics may be an essential trigger or driver of replay in other regions. To test whether the hippocampus is required for procedural memory formation in our task, we performed large bilateral lesions to the hippocampus via injection of a virus that expresses caspase (Fig. 5a, Extended Data Fig. 9 and Methods). Despite these large lesions, we could not detect a difference in the learning between hippocampus-ablated mice when compared to controls (Extended Data Fig. 9f–o). Lesioned mice reached the final task level in an equivalent number of trials and their learning curves were not significantly different from controls (Fig. 5b). After learning (trials 4,000–5,000), mice completed the task with comparable movement speeds (Extended Data Fig. 9f) and made a similar number of port-to-port transition errors (Extended Data Fig. 9g). Also, like control animals, lesioned mice were highly task focused, rarely poking into task-irrelevant ports (Extended Data Fig. 9i). In sum, we found that mice with hippocampal lesions were indistinguishable from controls across all measures of task performance.

Given that procedural consolidation is independent of the hippocampus, a compelling hypothesis is that the hippocampus is not involved in shaping replay of procedural activity in the striatum. To test this, we performed large bilateral lesions of the hippocampus and then recorded neural activity in the DLS using implanted Neuropixel probes (Fig. 5c). Just as for baseline recordings (nonlesioned mice), applying PP-Seq to post-task sleep epochs revealed awake-like neural sequences replayed offline (Fig. 5d,e). Across all measures, we could not detect any major differences in the characteristics of these events after hippocampus lesions. As observed in control recordings, neural sequences were also ordered with respect to awake sequences at the single-neuron level (Fig. 5f). At a macro-scale, sequences were also still replayed individually and in combination (Fig. 5g,h). When neural sequences were replayed in combination, they were still more likely to occur in the order in which they occurred during the behavioral sequence (Fig. 5i). As with control mice, we could not detect a difference in the proportion of forward and reverse replay in the lesion mice and replay progressed at the same range of speeds in the control and lesioned mice (Fig. 5j). We were also unable to detect a difference in the rate, length, extent or decay of replay events between groups (Fig. 5k,l,m and Extended Data Fig. 9). As in control mice, neurons that most consistently contributed to the awake neural sequence were still more likely to occur in replay in the lesioned mice (Fig. 5m,n and Extended Data Fig. 9q,r). This suggests that, at the single-neuron level, cells that consistently contributed to the awake sequence were still prioritized for replay. Together, this suggests that the hippocampus has no role in generating striatal replay in temporally compressing the replay, compositionally ordering the replay or prioritizing the replay at the individual neuron level.

**Extended Data Fig. 10:**
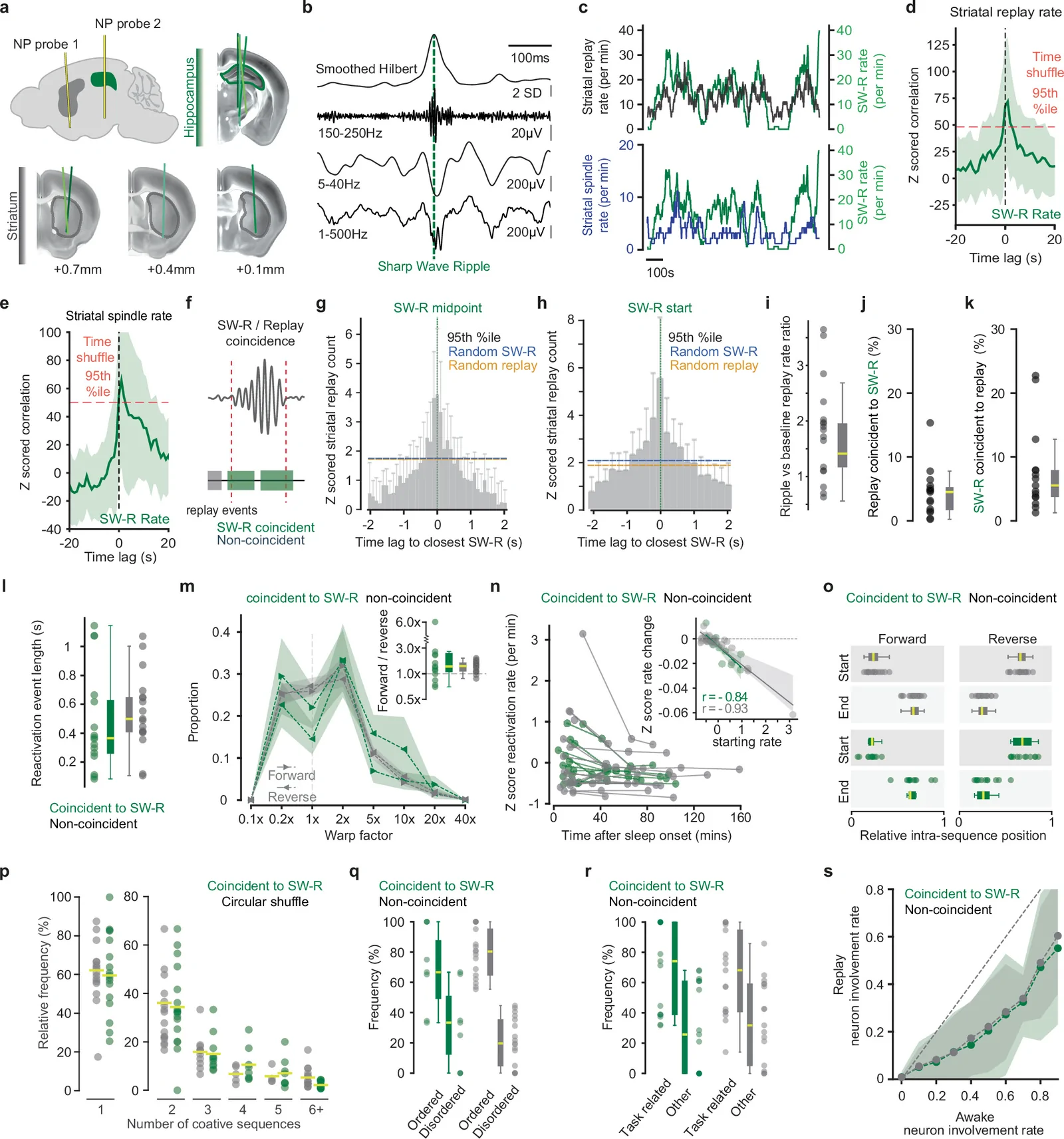
Relationship between replay and SW-Rs. **a**. Top Left: schematic illustrating dual Neuropixel probe implant approach. Top Right: implanted probe locations (probe midline) in hippocampus projected onto standard Allen atlas in median coronal plane. Bottom: Same for striatum implants. **b**. Example identified sharp-wave ripple. Events were detected with cross band filtering (see methods for details). **c**. Top: Example rate plots showing sharp-wave ripples per minute against striatal replays per minute (1 s increment bins, 20 bin smoothing window). Bottom: Same as above but showing example sharp-wave ripple rate against striatal sleep spindle rate. **d**. Cross-correlation between smoothed Sharp-wave ripple rates and striatal replay rates (Pearson correlation coefficient as a function of time lag. Red dotted horizontal line represents the 95th percentile of shuffled data from randomly permuting sharp-wave ripple time bins.) **e**. Same as figure d. but showing the relationship between sharp-wave ripple rates and striatal spindle rates. **f**. Schematic showing coincidence classification between striatal replay and sharp-wave ripple events. **g**. Mean binned distance to closest sharp-wave ripple midpoint for each striatal replay. Dotted lines represent the 95 h percentile for random data for randomly selected replay (orange) and Sharp wave ripple (blue) times. Error bars are standard deviation **h**. Same as g but for sharp-wave ripple start times. **i**. Ratio of replay occurring inside sharp-wave ripple events to replay outside of spindles. Normalised by time in each state. **j**. Percentage of all striatal repay events that were coincident (overlapping) of a Sharp-wave ripple. **k**. Percentage of all sharp-wave ripple events that were coincident (overlapping) of a striatal repay event. **l**. Mean replay event lengths (first to last spike duration) for sharp-wave ripple coincident and non-coincident striatal replay events (two-sided Wilcoxon rank sum, p = 0.231, Rank-Biserial r = 0.35). **m**. Main: distribution of warp factors (1x represents awake speed) for forwards and backwards replay events (Coincident vs Non-coincident: PERMANOVA; p = 0.509, R^2^ = 0.75). Inset: proportion of forward vs reversed replays (1x represents equal proportion. Coincident vs Non-coincident; two-sided Wilcoxon rank sum; p = 0.489, Rank-Biserial r = 0.22). **n**. Main: Z-scored reactivation rates for each analysed sleep epoch against time from first sleep onset. (Multivariate comparison between groups, MANOVA; Wilks lambda, p = 0.989, η^2^ = 0.0005). Inset: rate change against starting rate for each pair of epochs per session (Coincident; OLS; r = -0.84, R^2^ = 0.70, ***p < 0.001. Non-coincident; OLS; r = -0.933, r^2^ = 0.87, ***p < 0.001). **o**. Mean start and end points for all forward (left) and reverse (right) SW-R linked and unlinked replay events. Position is relative to the corresponding average awake sequence (MANOVA; Wilks lambda, p = 0.752, η^2^ = 0.09). **p**. Frequency of single (isolated) and concatenated events for each recording session for SW-R linked replay vs replay linked to event times drawn from circular shuffle of the SW-R event times (see methods. PERMANOVA; p = 0.899, R^2^ = 0.78). **q**. For sessions with coactive replay sequences; relative frequencies of task ordered and disordered concatenated sequences with respect to observed awake task ordering. Recordings contained 4 or more task related sequences (see methods. Multivariate comparison between groups, PERMANOVA; p = 0.304, R^2^ = 0.58) **r**. Normalised percentages of task and non-task related replay observed (Multivariate comparison between groups, MANOVA; Wilks lambda, p = 0.642, R^2^ = 0.61). **s**. Relative individual neuron involvement frequencies for each sequence during awake task activity and sleep periods (Multivariate comparison between groups, MANOVA; Wilks lambda, p = 0.939, η^2^ = 0.01). Panels d,e,g-s; n = 17 sessions, n = 4 mice. Boxplots show median IQR and 1.5 IQR, Displayed stars: *p < 0.05, **p < 0.01, ***p < 0.001. Source data

Although these results demonstrate that the striatal replay is generated independently of the hippocampus, these events may still be coordinated globally with replay in other regions such as the hippocampus^34,40,42^. To test this, we simultaneously recorded striatal replay and hippocampal SW-Rs, large amplitude oscillatory events that are correlated with replay in the hippocampus (Extended Data Fig. 10a,b). As has been shown for cortical reactivations^34,40,42^, striatal replay was correlated with an increase in SW-Rs in the hippocampus (Extended Data Fig. 10c–h). In line with this, there was an increased rate of replay during SW-Rs (Extended Data Fig. 10i). However, only a small percentage of striatal replay events occurred coincidently with SW-Rs and the minority of SW-R events were coincident with striatal replay (Extended Data Fig. 10j,k). This indicates that, although the striatal replay events can be coordinated globally with other reactivation events, most of the time they occur independently from SW-R events in the hippocampus. In line with our hippocampal lesion results, we could not detect any difference in the features of replay between striatal replay that was coincident and striatal replay that was noncoincident with SW-Rs (Extended Data Fig. 10l–s).

## Discussion

Our results demonstrate that sequences related to procedural experience are replayed in the dorsal striatum during sleep. As with replay in other areas such as the hippocampus, striatal procedural replay occurs at both real-world and time-compressed speeds^33^, occurs in both forward and reverse directions and is ordered compositionally in a manner consistent with awake experience. Task-related sequences linked to salient behavioral events are preferentially replayed in the forward direction, with reward and error outcomes differentially shaping replay content. Replay of reward-associated sequences incorporated more consistently task-active neurons, whereas error-associated sequences show the opposite pattern. Subsequent task improvements were predicted by forward replay events that contained consistent task-active neurons. Remarkably, these striatal replay features emerged independently of hippocampal activity, revealing an autonomous consolidation mechanism for procedural memory.

Previously, it was proposed that the hippocampus may be critical for the active consolidation of both hippocampus-dependent declarative memory and memories that do not require the hippocampus for expression, such as procedural memory^2,3,6–8,13,43^. The theory is that replay of a spatiotemporal context in the hippocampus triggers the active consolidation of all kinds of memory by boosting context-related representations throughout cortical and subcortical circuits^3,6,43^. In contrast to this theory, our results show that the hippocampus is not involved in either the generation of replay in the dorsal striatum or the consolidation of our procedural memory task. Our results support the alternative idea that there are ‘parallel memory systems’^19,22,44^ where consolidation can occur independently. We suggest that, within the parallel memory systems, replay may be a common mechanism for active consolidation. This suggests that either there are different sources of replay for distinct types of memory or there may be a common unappreciated source for generating replay that is independent of the hippocampus. Although our results support a body of literature that has shown that procedural memories can be formed independently of the hippocampus^16–18,20,21,44,45^, others have shown that the hippocampus can in specific situations support the sleep-dependent consolidation of procedural memory^5,8,11,15,46,47^. In these latter cases, it may be when the spatiotemporal context or some other hippocampus-dependent computation is important for learning^11,46^. Together our results support the theory that declarative and nondeclarative memories can be consolidated in parallel, but show that replay may be a common mechanism for active consolidation of both types of memory.

If replay is a universal mechanism for memory consolidation, then what is the mechanism for driving replay if it is not the hippocampus? One option is that the hippocampus still generates replay for declarative memories and there is a different source for replay in circuits related to procedural and other types of nondeclarative memory. However, even in cortical–hippocampal loops there is some evidence that patterned activity in the cortex precedes and predicts the content of replay in the hippocampus^41,48–50^. This suggests that replay may originate in parallel in both the cortex and the hippocampus. We suggest that it needs to be considered that replay is a phenomenon that occurs spontaneously in cortical and potentially other networks and that there might not be a single source.

### Unsupervised methods may help uncover the source of replay

Whether or not there are specific circuits that generate replay, we propose that the use of unsupervised methods such as the one that we adapted here will aid in the investigation of replay. One advantage of these approaches is that they reduce the a priori assumptions about how replay should progress, such as that sequences of activity progress in a linear manner (for discussion see refs. ^31,33^). These assumptions have been shown to limit the types of replay that are identified. When recent methods that removed the assumption about the constant speed of replay were developed, hippocampal replay was found to progress at a range of speeds, including real-world speeds^33,46^, just like the striatal replay that we identified in this paper. Unsupervised approaches can also be used to examine replay without the generation of a behavioral template^51,52^; the weakness of this is that these approaches can identify neural sequences that are not related to the behavioral features of interest. This might make these approaches better suited for tasks with repetitive structure, like ours. When these methods are applicable, we show that they can in practice be more sensitive and accurate than even state-of-the-art decoding methods.

### Striatal replay

Our discovery that replay occurs in the dorsal striatum independently of the hippocampus is consistent with previous work showing that there is reactivation of task-related ensembles in the dorsal striatum after motor skill learning and that this occurs in combination with an increased synchrony between cortical and striatal ensembles^28,53^. We now show that the features of this reactivation mirror those associated with replay in the hippocampus and cortex. This sets the stage for investigating the role replay plays in a host of dorsal striatum-dependent tasks, from perceptual learning to value-based decision-making^54–56^. It will be of particular interest to determine how striatal replay is coordinated with dopamine release because both phenomena are strongly linked to memory formation. Indeed, in quiet wakefulness, reward-related neurons in the ventral tegmental area, putatively dopamine neurons, are coordinated with hippocampal replay, but this is not the case during sleep^57^. Despite this, dopamine has been shown to have a causal role in offline memory consolidation^58–61^ and whether this is due to coordination between replay and dopamine release in the dorsal striatum needs to be investigated.

Currently, we have shown that offline processing in the striatum is needed for procedural memory consolidation and suggest that replay might be the process that drives this active consolidation. To prove this, it will be important to selectively disrupt striatal replay and assess the impact on memory formation. A similar approach is possible in the hippocampus because replay is believed to occur almost exclusively during SW-Rs^34^, although others have questioned this assumption^62,63^. Due to this, closed-loop inactivation of the hippocampus during ripples has been used as a proxy for disrupting replay and showing that it causally contributes to consolidation^64,65^. Without a biomarker for striatal replay, a similar approach will not be possible; new approaches may need to be developed to rapidly detect the onset of replay to target these patterns for disruption. This would allow targeted disruption of replay to determine the functional contribution that replay makes to learning, consolidating and retrieving procedural memories.

### Summary

We show that replay in the striatum occurs during offline consolidation of procedural learning through a hippocampus-independent mechanism. Replay content is shaped by behavioral outcomes and predicts subsequent performance improvements. These findings reveal that replay is a universal mechanism for active memory consolidation during sleep, but that distinct memory systems generate replay independently. Future work will be needed to identify the sources of hippocampus-independent replay and determine how replay in distinct networks is coordinated to support integrated memory consolidation.

## Methods

### Animals

Adult male and female C57BL/6J mice (aged 8–50 weeks; wild-type) were used. Mice were housed in HVC cages with free access to chow and water on a 12 h-to-12 h, inverted light-to-dark cycle and tested during the dark phase. Mice used in sleep-recording experiments were housed on a noninverted light-to-dark cycle and tested during the light phase (normal daylight hours). For behavioral experiments, mice were water deprived. Animals had access to water during each training session and, otherwise, water was administered by hand. Water was supplemented as needed if the weight of the mouse was <85%. All experiments were performed in accordance with the UK Home Office regulations, the Animal (Scientific Procedures) Act 1986 and the Animal Welfare and Ethical Review Body. Animals in test and control groups were randomly selected.

### Behavioral procedures

Mice were trained once a day, in sessions that lasted ~1 h (mean session length = 69.04 ± 20.51 min, *n* = 83 mice). Training was conducted in custom-built behavioral arenas measuring ~16 cm × 19 cm × 24 cm (width, length, height). Box walls were made of 0.5-cm-thick, opaque white or transparent red acrylic and had eight poke ports mounted on the front wall. Ports (Sanworks, ID 1010) protruded 2 cm from the wall into the area and were arranged in a 4 × 2 grid such that neighboring ports (vertical and horizontal) were 3 cm apart (centroid to centroid). Ports contained a side-mounted infrared light-emitting diode (LED) and sensor to detect poke events and a back-mounted visible light LED to illuminate the port. Each port also contained a waterspout for reward delivery. Poke events were registered by Sanworks port PCBs (ID 1004) connected to a Bpod (ID 1027) programmed with a customized behavioral protocol (MATLAB). A 2-μl water delivery system was triggered by Bpod via a connected Miniature Solenoid (Lee Co., cat. no. LHDB0513418H). Sounds were played at port entry via a speaker (DigiKey, part no. HPD-40N16PET00-32-ND) and amplifiers (DigiKey, part no. 668-1621-ND).

Mice were rewarded for completing the full five-step poke sequence. No reward was given if animals missed a step in the sequence, but animals were not punished for adding extra steps into the sequence. Single-trial events were defined as all poking activity that led to reward delivery at the final port. Within a single trial, animals could make multiple attempts at completing the sequence or add additional elements to the sequence and still eventually receive a reward. The task was self-paced, although, if an animal initiated a trial but did not register a poke into any port for 30 s, this trial timed out, was left unrewarded and a new trial was cued. Across all training levels, when animals entered a port (breaking the infrared beam) a short-duration sibilant noise was played.

Poke sequences were shaped by an automatic protocol with 50 training levels of predefined difficulty. Mice started from the lowest level (level 1) and progressed up to the final task (level 50) (Extended Data Fig. 1a,b). During training, performance was assessed every ten trials and this metric determined whether mice progressed up a level (performance >90%), regressed down a level (performance <20%) or remained at the same training level (20% < performance < 90%). In early task levels (1–12), mice were rewarded for performing each step of the five-step sequence. With progression to higher levels, reward steadily decreased and then switched off port by port until reward was given only on reaching the final port (levels 12–50). In early task levels (1–12), each step in the sequence was also visually guided by successively illuminating port LEDs which were switched off port by port once the animal had successfully poked. After this (levels 12–49), across successive levels LED brightness was gradually dimmed port by port and eventually turned off permanently, except for the initial port in the sequence, which remained illuminated at the start of each trial to signal that a new trial was available. At the final stage of the task (level 50), only the first port in the sequence was illuminated in this way. Even after reaching the final task (level 50), during training animals could drop down to lower levels if they performed badly. However, in circumstances where it was necessary to test animal performance at the full task, mice were held at level 50 for the duration of the session. For AP5 infusion experiments, to increase sensitivity to performance changes during test sessions, training performance was assessed (and the training level updated) every four trials. For hippocampus lesion experiments, animals were trained on the task for at least 5,000 trials, except for 1 animal which was only trained for 2,004 trials. This animal was excluded from analysis of post-learning performance for this reason.

### Surgical procedures

Mice were anesthetized with Isoflurane (0.5–2.5% in oxygen, 1 l min^−1^)—also used to maintain anesthesia. Carpofen (5 mg kg^−1^) was administered subcutaneously before the procedure. Craniotomies were made using a 1-mm dental drill (Meisinger, cat. no. HP 310 104 001 001 004). Injections were delivered using pulled glass pipettes (Drummond, 3.5 inches) on a stereotaxic frame (Leica, Angle Two).

For striatal lesions, initial lesions were excitotoxic via injection of NMDA (2 mg per 100 ml), although for most animals shown lesions were achieved by injecting a 1:1 mix of AAV2/1-hSyn-Cre (10^14^ vector genomes (vg) ml^−1^) and AAV2/5-EF1a-DIO-taCasp3-T2A-TEVp (10^14^ vg ml^−1^). The mix was diluted 5× in saline buffer before injection. For control animals, saline or GFP virus AAV2/5-CAG-EGFP (10^12^ vg ml^−1^) was injected instead. In each hemisphere, four injections (~80 nl each) at three different depths were made to distribute the viruses as evenly as possible and, to provide enough coverage, injections were targeted to the DLS or DMS dependent on the experimental group. For DLS, injections were made at coordinates anteroposterior (AP) 0.2–0.8 mm, mediolateral (ML) 2.5–2.7 mm and dorsoventral (DV) −3.0 mm to −3.7 mm (where a range is given, injections were given at regular spacing between these values). For DMS, insertions were made at coordinates AP 0.2–0.8 mm, ML 1.8 mm and DV −3.0 mm to −3.7 mm.

For hippocampus lesions, the same Cre-caspase mixture used in the striatal lesion experiments was injected. Control animals underwent the same surgical procedures, except saline was injected instead. Injections were made bilaterally at 26 locations per hemisphere (Supplementary Table 1). After surgery, animals were given at least 3 weeks of recovery before training was started.

For cannulation experiments, 5-mm 26G cannulas (P1 technologies, cat. no. C315GS-5/SPC) were implanted at coordinates AP 0.5 mm, ML 2.9 mm and DV 2.0 mm from the pial surface, with a 10° tilt (tip tilted toward midline). For optogenetic experiments, we implanted flat optical fibers of 200-μm diameter (Newdoon, cat. no. FOC-C-200-1.25-0.37-7) at AP −1.6 mm, ML −1.9 mm and DV 2.8 mm (20° ML tilt). Implants were affixed using light-cured dental cement (3M Espe Relyx, cat. no. U200) and dental cement (Super-Bond C&B Bulk-mix, Sun Medical) and the wound sutured (6/0, Vicryl Rapide).

For Neuropixel implantation, before training animals underwent an initial surgery in which the skull was exposed, coated with a thin layer of dental cement and marked for later skull leveling. A craniotomy was drilled over an arbitrarily chosen region of posterior cortex and a ground pin was implanted. To replicate the weight and size of the eventual implant, mice were trained on the task with a size-matched and weight-matched dummy implant, which was fixed during initial surgery to the cement layer using silicon (Kwik-sil, World Precision Instruments). For probe implantation, the dummy implant was removed and the skull leveled using previously noted skull markings. For striatum implants, a craniotomy and durotomy were made at coordinates AP 0.8 mm and ML 2.1 mm and the probe was implanted to a depth of 4.0 mm at a 10° angle (tip tilted away from the midline). For hippocampal recordings, craniotomy and durotomy were at AP −2.5 mm and ML −1.3 mm and the probe was implanted straight, to a depth of 4.0 mm. External grounding wires were fixed to the previously implanted skull pin. Craniotomies were sealed using Duragel (Cambridge Neurotech) and a three-dimensional (3D) printed casing was fitted around the probe for protection. Implants were performed using a retrievable system^66^ and, in some cases, animals were reimplanted with a second probe. At the end of the experiment, probes were recovered for future reuse.

### Electrophysiological recordings

Animals were habituated to the size and weight of the implant by first training on the task with a size-matched and weight-matched dummy implant fixed to the skull. Dummy implants were constructed from a 3D printed plastic casing, aluminum implant cassette and surgical tape. During training, to simulate eventual recording conditions, animals were tethered to an overhead cable connected to a motorized rotary joint (Doric, cat. no. B330-1027-001). Mice were also habituated to sleeping in their home cage (roof open) while connected to this tether. To increase sleep incidence during recordings, all mice (except for one) were housed with a normal light-to-dark cycle. Neuropixel 1.0 or 2.0 four-shank probes were implanted through the motor cortex and striatum or hippocampus and, after implantation, daily recording sessions were conducted as continuous recordings across sleep and task epochs (3–6 h). These recording sessions usually consisted of three stages. First, animals were placed in their home cage and recording commenced once the mouse fell asleep. After roughly 1–2 h of pre-task sleep, mice were then woken and placed into the behavioral arena to complete the task. Finally, animals were placed back into the home cage and left for several hours to capture post-task sleeping. Recordings were acquired using Neuropixels acquisition hardware (imec Neuropixels 1.0 or 2.0 headstage, interface cable and PXIe Acquisition Module) with open-Ephys software. Post-acquisition spike sorting was done using Kilosort3^67^. Spike sorting was checked using Phy2 (https://github.com/cortex-lab/phy/) but analysis was not performed on manually curated clusters.

### Pharmacological manipulations

For muscimol infusions, ~30 nl of either muscimol (Sigma-Aldrich) at 0.2 mg ml^−1^ or saline buffer was infused via implanted cannulas. The infusion system consisted of a 1-µl Hamilton syringe (Merck), plastic tubing (P1 Technologies, cat no. 8F023X050P01) and injection cannulas (P1 Technologies, cat no. C200IS-5/SPC). Tubing was filled with mineral oil to ensure an air-tight setup for accurate volume administration. Animals were briefly head-fixed and infused at a rate of 10 nl min^−1^ for 5 min per cannula. Animals were then allowed to rest in their home cage for 10–15 min and tested on the task. Between muscimol infusion experiments, animals were given a recovery break of at least a day and task behavior was assessed on this break day to ensure that performance returned to that pre-infusion. All animals were habituated to head fixation before experiment onset.

For AP5 experiments, we adapted methods described previously^28^. We bilaterally infused 450 nl of saline or 450 nl of 5 µg µl^−1^ of D-AP5 (Bio-Techne, diluted in saline), via cannulae implanted into the DLS. Immediately after training, animals were head fixed and infusions were carried out at a rate of 65–90 nl min^−1^ for 5–7 min per cannula. After infusion, animals were returned to their home cage. In the test session, the next day (~24 h later) animals were trained on a performance-sensitive version of the behavioral task (see ‘Behavioral procedures’ section). This allowed for higher sensitivity in detecting changes to task performance. Infusions during learning were done from level 12 to level 49 (after the reward-guided habituation phase: levels 1–11). Infusions were done if animals climbed at least three levels that session but were not done on consecutive days. Before the experiment, animals were habituated to head fixing. Infusions of saline and AP5 were alternated throughout the learning curve of each animal. After learning, once animals reached stable expert performance (level 50) for at least 4 d, infusions of either saline or AP5 were given for 4 d consecutively. All mice were used for both experimental groups and, so, before switching the type of infusion, animals were trained until at least 4 d of stable expert performance was seen. One animal was unable to reach level 50 with stable performance, so was excluded from this experiment.

### Tissue processing and image analysis

At the end of experiments, animals were euthanized via intraperitoneal injection (10 ml kg^−1^ of pentobarbital) and brain tissue fixed via vascular perfusion (4% paraformaldehyde) and collected for histology. Before implantation, Neuropixel probes were coated in dye (DiI) for later visualization. Brains were imaged using serial section two-photon imaging^68^. Our microscope was controlled by ScanImage Basic (Vidrio Technologies) using BakingTray, a customized software wrapper for setting up the imaging parameters (https://github.com/SainsburyWellcomeCentre/BakingTray, https://doi.org/10.5281/zenodo.363160) and nine images were assembled using StitchIt (https://github.com/SainsburyWellcomeCentre/StitchIt and https://zenodo.org/badge/latestdoi/57851444). The 3D coordinates of the injections, fiber, cannula and Neuropixel probe placements were determined by aligning brains to the Allen Reference Atlas (Mouse Brain: available from atlas.brain-map.org) using brainreg^69^ and the tracks were located and traced in common atlas coordinates (brain-reg segment).

Brain slices were all stained following the same procedure: blocking in staining solution (phosphate-buffered saline + 1% bovine serum albumin + 0.5% Triton-X) for 1 h: primary antibody(ies) (1:1,000 in staining solution) for 2–4 h at room temperature or overnight at 4 °C while rocking and a 15-min wash with staining solution; and secondary antibody(ies) (1:1,000 in staining solution) and DAPI for 2 h at room temperature while rocking. Slices were then washed in phosphate-buffered saline and mounted using Prolong or ProGold mounting medium (Thermo Fisher Scientific). Primary antibodies used were NeuN (abcam, cat. no. ab177487) and glial fibrillary acidic protein (abcam, cat. no. ab4674). Secondary antibodies used were Alexa Fluor-488 anti-chicken (Thermo Fisher Scientific, cat. no. A-11039) and Alexa Fluor-647 anti-rabbit (Thermo Fisher Scientific, cat. no. A-21245).

For striatal lesions, brains were sliced using a cryotome at a thickness of 40 μm and with a vibratome at 100 μm for hippocampal lesions. Then, 15–20 slices covering the entire region at regular intervals were selected for NeuN and glial fibrillary acidic protein staining. Slices were mounted in standard glass slides using standard mounting medium and subsequently imaged in the Slide Scanner (Zeiss) using a ×20 objective. The lateral and medial striata were defined by the extent of axons from prelimbic or cingulate projections and motor cortical projections, respectively (Allen projection experiment nos. 157711748, 112514202, 180720175 and 180709942). The lesioned areas for each of these regions was determined manually for each slice using the brain-reg segment. Mice with lesions that had >20% of volume overlapping with the cortex were excluded from the analysis.

### Performance measures

A trial was defined as all the poke events that occurred after reward or trial time out (no pokes for 30 s). Performance was calculated per trial and involved segmenting sequence pokes into ‘attempts’ that were temporally relevant (within 2 s of each other). Attempts were considered as starting only from the initiation port (port 1); any pokes that occurred before the first poke into port 1 were excluded. An attempt was assigned a value of 1 if it contained the perfect poke sequence and 0 if it contained an error. The mean score across these attempts was then calculated. During training, a simplified measure was used to calculate ongoing trial-by-trial task performance used for updating training level; rather than across ‘attempts’, the same measure was calculated across trials. Ongoing performance was scored as a mean over a window of ten trials except for AP5 test sessions where this window was reduced to four trials.

### Video-tracking analysis

Videos were captured at 60 frames per s (Telendyne Flea3) from an elevated, down-angled position behind mice, in line with the task port wall. Mouse movements were tracked using DeepLabCut^70^. Tracking points <98% CI were excluded and replaced by interpolating between accepted points. Task movement variability was first calculated individually for different task subsequence (movement vectors). To achieve this, only trajectories that passed close to each port in the subsequence (within a 1-cm radius) in order and with appropriate timing (within a 2-s port-to-port time window) were considered. Trajectories were averaged to find the mean trajectory curve. This curve was then segmented into 3,000 spatial bins to be used as a reference and Hausdorff distances were calculated. These distances were then used to calculate the s.d. (movement variability) of each tracked trajectory from the mean curve. To create the standard space sequence occurrence plots during analysis of PP-Seq output data, a similar analysis was done. However, average trajectory curves were generated for the entire task sequence rather than individual subsequence chunks. PP-seq labeling of averaged tracking data was based on the mean ‘dominant’ (most often observed) sequence type that occurred for spatial bins across trials. When labeling continuous tracking across multiple trials by PP-seq sequence, tracking was split into 200-ms time bins and labeled by the sequence in each bin that contributed the most labeled spikes.

### PP-Seq replay detection

We adapted PP-Seq^35^ such that, after training the model on a set of awake data, the parameters that defined each neural sequence could be fixed per their fit from the awake data. This allowed us to then apply each trained model to sleep data, to search for the same recapitulated neural sequences. Our adapted model can be found here: https://github.com/lindermanlab/PPSeq.jl/pull/15.

PP-Seq is a generative probabilistic model for identifying sequential neural activity. It models neural firing as an inhomogeneous Poisson point process, such that firing rate changes are a function of a latent sequence variable that captures shared temporal structure across neurons. Model fitting is done through an iterative sampling procedure, collapsed Gibbs’ sampling, that converges to drawing samples from the posterior distribution on model parameters given the data (full detail in ref. ^35^). The objective function corresponds to the negative log(likelihood) of the observed spike trains under the generative model, which is minimized to obtain the best probabilistic explanation of the data.

Running PP-Seq required setting 12 hyperparameters. The three values related to background firing were set in accordance with the reported literature, as were those related to the width of a neuron’s response. Of the seven remaining hyperparameters, five were chosen by grid search (Supplementary Table 2 and Supplementary Fig. 2a–f), the other two were found in preliminary work to have little effect on the overall PP-Seq output. We used crossvalidation to train and test the model: a subset of spikes was held out from the data, the rest used to train the model, then the log(likelihood) of the held-out spikes was measured under the trained model. The hyperparameters that led to the highest held-out log(likelihood) were taken as those that best capture the structure in the data and could predict held-out spikes. We chose our selected model from the top 20 models (all within error of each other) by visual inspection of the output labeling. Together, this specified the 12 hyperparameter values that we used for our subsequent analysis. We performed this hyperparameter fitting on the data from one animal, then used the same values, occasionally scaled for the number of neurons and average firing rate in the data, for all other mice.

The PP-Seq algorithm was then applied to each recording session individually. Striatal neurons were first filtered to remove high and low firing rate units (Fano factor 0.5–12) and a 600-s to 1,000-s period of high task engagement awake activity was chosen on which to fit the model. To sample replay during our offline recordings, PP-Seq was applied to segments (500–1,500 s) of the recording totaling at least 2,000s throughout the sleep period. We permitted each model to fit an additional two latent sequences and, because PP-seq directly infers the most likely time warping value for identified latent events, we utilized this feature to identify replay at different speeds.

After running PP-Seq, the neural sequences in awake and sleep recordings were pre-processed before further analysis was performed. As PP-Seq is a probabilistic model, its outputs represent a posterior over the assignments of the spikes to each latent, in the form of many samples from the posterior. We made use of this to filter for only high-confidence assignments, by analyzing only spikes that were consistently assigned to a sequence with 75% probability. Single replay events were defined by binning PP-Seq-labeled spikes into 20-ms time bins and grouping bins together if they were adjacent and contained spikes for a given sequence type. Replay instances were analyzed only during classified sleep periods and replay events were then excluded from analysis if they did not contain at least seven spikes from five neurons. Coactive replay events were defined as any sequence of events that occurred within 300 ms of each other. Replay events were filtered further by regression analysis of their progression with respect to average awake sequential position. Regression slopes were used to define warp factor for each replay event. For analysis of coactive sequence ordering, ordered coactive pairs of sequences were those that respected observed task ordering (forward or reverse) or pairs that were a repeat of the same sequence type. Disordered sequence pairs were those that did not normally occur adjacent to each other in the task sequence (forward or reverse). Recordings with only three task-related sequences were excluded from this analysis because they could not contain disordered pairs. To determine replay propagation extent compared to awake sequences, neuron-to-neuron spike ordering for each event was compared to the expected awake ordering for that event type.

### Bayesian decoders

The state–space decoder (decoder 1) used was as described in ref. ^33^. Models were trained for each recording session to predict a 2D posterior (video-tracking position) from spiking data. The linear decoder (decoder 2) used was as described in ref. ^39^. Models were trained for each recording to predict a 1D posterior (linearized task space taken from average video-tracking position). Training data for each model were filtered to include only successful movement trajectories. Filtering was done spatially: only trajectories that passed close to each port (within a 1-cm radius) in order and with appropriate timing (within a 2-s port-to-port time window) were considered. Spatial bins were 3× the average distance traveled in one-time bin (20 ms); on average this corresponded to ~400 spatial bins.

Replay detection was done by applying the trained models to short data segments of interest; usually 1–5 s of data. For decoder 2, detected events were defined as true replay or noise by quantifying the spatial coherence of the decoded position. This was defined by whether the number of spatial bins necessary to explain the prior position up to a 95% CI was within a threshold value. Thresholds were calculated before applying the decoder to sleep data. For each event type in each recorded session, the threshold number of bins was calculated from the distribution of 95% posterior density for >200 awake events and periods of random noise activity. Based on these distributions, the threshold value was set to maximize the number of true positives while minimizing the number of false-positive events.

For decoder 2, replay was assessed as described in ref. ^39^. Putative replay events were first scored using a line-fitting algorithm and events with a reasonable linear fit were then assessed for significance through a shuffle analysis. Both of these analyses depend on a width parameter, which scales the degree of variation from the linear fit that is accepted. This parameter was set dynamically for each model by comparing the rate of true-positive and false-positive synthetic replays found. The chosen parameter for each model was that which maximized true replay detection while minimizing false positives.

### Synthetic data testing

The synthetic replay data were generated by implanting PP-Seq-detected sequences into background noise. Background noise was generated by randomly permuting neuron IDs from awake activity. Hence, spiking content in background noise and the original spike data were identical, but the neuron IDs given to PP-Seq were shuffled. For each test, sequences were extracted from the corresponding PP-Seq-labeled awake dataset by manually setting an inclusion zone generated by a time window centered on the middle of the detected sequences. All spikes in each inclusion zone were extracted. Sequences were then filtered for representative, noncontaminated sequences. This was done by excluding the top and bottom 25% of sequences based on total sequence spikes and the number of contaminant (other sequence) spikes in these windows. Sequences that did not occur regularly in the labeled data were excluded from this analysis. From all extracted sequences, 200 sequences were then chosen (selected to maximize equal numbers from all sequence types extracted) for implantation into 600 s of noise. When required, sequences were then manipulated and altered. For warp values that stretched the sequences, <200 were implanted to avoid excessive overlap between sequences. In rare cases where <200 sequences were originally extracted in total, then random sequences from underrepresented groups were duplicated to make up this number. The chosen sequences were then randomly ordered and implanted into noise roughly equally spaced apart. Noise data were deleted where sequences were implanted such that the implanted sequences replaced spikes in these regions. For PP-seq testing, replays were considered correctly labeled if they were identified as the same sequence type that was inserted in the synthetic data; otherwise they were deemed mislabeled. For decoder testing, events found to have a spatial overlap of at least 40% with the original inserted replay location were considered as correctly labeled events; those that didn’t were deemed mislabeled.

### LFP analysis

Local field potential (LFP) was downsampled to 2.5 kHz from each 30-kHz Neuropixel channel. For sleep state classification and spindle analysis, data were then low pass filtered at 100 Hz (fourth-order Butterworth filter). For SW-R analysis, this initial low pass was at 1,000 Hz.

For sleep state scoring, REM and NREM periods were classified from power spectral density of the LFP and movement based on methods described previously^28^. During recording sessions, neural signals were captured during 1-h-long to 4-h-long pre-task and post-task sleep periods. Video was captured at 60 Hz from a camera (Telendyne Flea3) placed directly above the home cage. Processed LFP from several (three to six) evenly spaced striatal electrodes along the Neuropixel probe were *z*-scored and averaged to generate a mean LFP; noisy electrodes were manually excluded. LFP and tracking data were split into nonoverlapping 20-s time windows. Movement was determined from video tracking. Processed, *z*-scored tracking points were averaged and used to determine mean movement velocity for each time window. Movement velocity below a threshold (0.8 *σ*) was classified as putative sleep. Each time window was then assessed for sleep state based on δ spectral power (1–4 Hz) and θ broadband ratio (5–10 Hz and 2–15 Hz, respectively), calculated using the multitaper particle size distribution method from the MNE-Python package. Epochs with high δ (above mean) and subthreshold movement were classified as NREM. Epochs with low δ (below mean), high θ ratio (above mean) and subthreshold movement were classified as REM. All other epochs were classified as awake. NREM and REM epochs that were <60 s long (three consecutive windows) were reclassified as awake.

To detect spindle events, we adapted a previously described approach^71^. Wide-band *z*-scored LFP was manually selected from at least three evenly spaced, noise-free channels in striatum. Channels were averaged and the resultant signal was filtered in the spindle band (9–16 Hz) using a zero-phase Butterworth band-pass filter implemented as cascaded sixth-order high-pass and eighth-order low-pass stages. We then computed a smoothed signal envelope as the magnitude of Hilbert convolved over a 200-ms Gaussian window. Spindles were defined as epochs in which this envelope exceeded an upper threshold (*μ* + 2*σ*) for at least one sample and exceeded a lower threshold (*μ*) for at least 300 ms. Where the envelope crossed the lower threshold defined spindle start and end points. To correlate replay rate with spindle and δ band spectral power, the LFP signals were segmented into windows of 1-s length, with an overlap of 0.5 s between consecutive segments. A Hanning window was applied to each segment to reduce spectral leakage. Fourier transform was computed using the fast Fourier transform (FFT) and the one-sided power spectral density was obtained by normalizing the squared magnitude of the FFT. Power was then integrated within the desired frequency band (9–16 Hz for spindles, 0.5–4 Hz for δ). In the crosscorrelation analysis, comparative phase shuffle was performed by taking the FFT, randomly shuffling the phase angles and then performing an inverse FFT to reconstruct the signal.

To determine the relationship between striatal replay and SW-Rs, we implanted two Neuropixel 2.0 probes, one in the DLS and another in the dorsal hippocampus. Recording sites in the hippocampus were initially chosen based on implant depth and LFP signatures, specifically θ band power^72^. Analyzed electrode channels were then selected post hoc based on the anatomical depth corresponding to the dorsal CA1 region and the presence of SW-Rs. To detect SW-Rs, the wide-band LFP signal of a subset of eight evenly spaced hippocampal probe channels was processed separately for ripple or sharp-wave extraction. For ripples, the signal was band-pass filtered (zero-phase IIR) between 150 Hz and 250 Hz and the envelope computed (absolute Hilbert), smoothed (Gaussian, *σ* = 12.5 ms), averaged across channels and *z*-scored. Event duration was determined by extending the interval from the peak to the nearest local minimum on either side. For sharp-wave detection, we band-pass filtered one LFP channel from the same subset between 5 Hz and 40 Hz and *z*-scored and inverted it. Sharp waves were defined as any threshold crossing of >3 s.d. Only if a sharp wave and ripple were simultaneously detected was an event retained for further analysis.

### Statistical analysis

A one-sample Student’s *t*-test (one group), paired Student’s *t*-test (two groups) or one-sample ANOVA (three or more groups) was carried out when the assumptions for a normal distribution of observation within groups (Shapiro–Wilks test) were satisfied. Otherwise, the nonparametric Wilcoxon’s signed-rank, Mann–Whitney *U*-test or Kruskal–Wallis test was used. When there were unequal observations between groups, an independent Student’s *t*-test was used, given that the assumptions were satisfied. When data (two or more groups) were multivariate, a MANOVA was performed (Wilks’ *λ P* statistic reported) or nonparametric PERMANOVA (pseudo-*F* *P* statistic after 10,000 permutations reported) for data that were not normally distributed. For multiple comparisons, post hoc tests used were Tukey’s HSD after ANOVA or MANOVA, post hoc Dunn after the Kruskal–Wallis test and pairwise PERMANOVA with Bonferroni’s correction after PERMANOVA. Test-relevant effect-size statistics are reported for each test. Statistical tests and effect-size statistics used were from the following Python packages: scipy.stats, statsmodels, scikit-bio, scikit-posthocs and pingouin. No statistical methods were used to predetermine sample sizes but our sample sizes are similar to those reported in previous publications^11,33,62^. Data collection and analysis were not performed blind to the conditions of the experiments.

For the statistical analysis of training learning curves, the data were downsampled into bins of 100 trials. Each animal’s learning curve was randomly reassigned to the lesion or control group to look at the mean difference between controls and lesions. This shuffling of learning curves was repeated 10,000× and all 10,000 means were used to find the 95% CI for the difference in the data due to chance. For comparison between task-related and nontask-related replay sequences, a similar permutation test was performed and repeated 10,000×. The resultant distribution of means was analyzed and the 95% CI reported. For analysis of coactive replay rates, expected percentages were calculated as the independent Poisson probability, given the mean rate of replay events (individual sequences) and a time interval of 300 ms plus average replay event length. For ordered and disordered replay analysis, the expected percentages, ordered and disordered, were calculated from the expected ratios of ordered and disordered given the number of task-related sequences in each recording. These expected ratios were summed across recordings to find overall expected percentages. For coactive analysis of replay linked to spindles and coincident to SW-Rs, to account for sampling bias during periods of higher replay rate, linked or coincident coactive replay was compared against replay, which was associated with the timepoints of spindle or SW-R events that were randomly shifted (circular shuffle).

To determine links between replay and behavior we performed a CCA. This multivariate procedure seeks linear combinations of feature sets *X* and *Y* that are maximally correlated across multiple orthogonal component dimensions. Mathematically, for canonical component *i*, this can be expressed:

$$\left({u}_{i},{v}_{i}\right)=\left({{\bf{a}}}_{i}^{{\top }}X,{{\bf{b}}}_{i}^{{\top }}Y\right),{\rho }_{i}={{\mathrm{corr}}}({u}_{i},{v}_{i})$$

where **a**_*i*_ and **b**_*i*_ are weight vectors defining canonical correlates, *u*_*i*_ and *v*_*i*_, which have a correlation (corr) *ρ*_*i*_. Feature variables were first normalized using Standardscaler from the preprocessing module of the Python package Scikit-learn; CCA was then performed using the crossdecomposition module. Resultant CVs were selected for further analysis based on the following procedure: first, the magnitude of the joint variation between variates given each canonical dimension was compared to shuffled data and a fivefold crossvalidated distribution. Variates for which the observed measures fell outside the 95th percentile of both null distributions were then further tested by comparing the linear correlation between variables under that canonical mode against the distribution of this measure in shuffle and crossvalidation conditions. As before, only variates for which the observed data fell outside the 95th percentile of both null distributions were analyzed further. To control for type I errors with multiple testing, *P* values were false discovery rate corrected. To assess the contribution of individual features to the CVs, we computed canonical weights. To estimate the stability of these weights, we performed bootstrap replacement resampling (1,000 iterations) recomputing the canonical weights for each resample. To estimate directional variance explained by each canonical mode, CCA redundancy was estimated by bootstrap resampling (1,000 iterations). For each resample, canonical correlations were computed and redundancy was quantified as the variance in one set predictable from the other.

### Reporting summary

Further information on research design is available in the Nature Portfolio Reporting Summary linked to this article.

## Online content

Any methods, additional references, Nature Portfolio reporting summaries, source data, extended data, supplementary information, acknowledgements, peer review information; details of author contributions and competing interests; and statements of data and code availability are available at https://doi.org/10.1038/s41593-026-02362-5.

## Supplementary information


Supplementary InformationSupplementary Figs. 1 and 2 and Tables 1 and 2.
Reporting Summary
Supplementary Video 1Video shows an example of expert task performance.
Supplementary Video 2Neural sequences shown in Fig. 2 aligned to behavioral performance.
Supplementary Video 3Example of a neural sequence aligned to a nontask event that is grooming.
Supplementary Data 1Source data for Supplementary Fig. 1.
Supplementary Data 2Source data for Supplementary Fig. 2.


## Source data


Source Data Fig. 1Statistical source data.
Source Data Fig. 2Statistical source data.
Source Data Fig. 3Statistical source data.
Source Data Fig. 4Statistical source data.
Source Data Fig. 5Statistical source data.
Source Data Extended Data Fig. 1Statistical source data.
Source Data Extended Data Fig. 2Statistical source data.
Source Data Extended Data Fig. 3Statistical source data.
Source Data Extended Data Fig. 4Statistical source data.
Source Data Extended Data Fig. 5Statistical source data.
Source Data Extended Data Fig. 6Statistical source data.
Source Data Extended Data Fig. 7Statistical source data.
Source Data Extended Data Fig. 8Statistical source data.
Source Data Extended Data Fig. 9Statistical source data.
Source Data Extended Data Fig. 10Statistical source data.


## Data Availability

The data that support the findings of this study are available via Zenodo at https://doi.org/10.5281/zenodo.20055819 (ref. ^73^). In addition, Allen Brain Atlas connectivity (https://connectivity.brain-map.org/) data were used to demarcate the corticostriatal projection patterns from prelimbic and cingulate cortex (experiment nos.: 157711748 and 112514202) and motor cortex (experiment nos.: 180720175 and 180709942). Source data are provided with this paper.

## Code availability

The analysis code is publicly available at https://github.com/StephensonJonesLab/Thompson_et_al_2026/tree/V1.02 and https://github.com/lindermanlab/PPSeq.jl/pull/15.
