# Supplementary material for: Replay of procedural memory is independent of the hippocampus
Source: Nat Neurosci. 2026 Jul 23;29(9):2237–48. doi: 10.1038/s41593-026-02362-5 (PMC13533836; doi:10.1038/s41593-026-02362-5)
Supplement: Supplementary file 1 [file 41593_2026_2362_MOESM1_ESM.pdf]

nature neuroscience

Supplementary information https://doi.org/10.1038/s41593-026-02362-5

# Replay of procedural memory is independent of the hippocampus

In the format provided by the authors and unedited

# Supplementary information

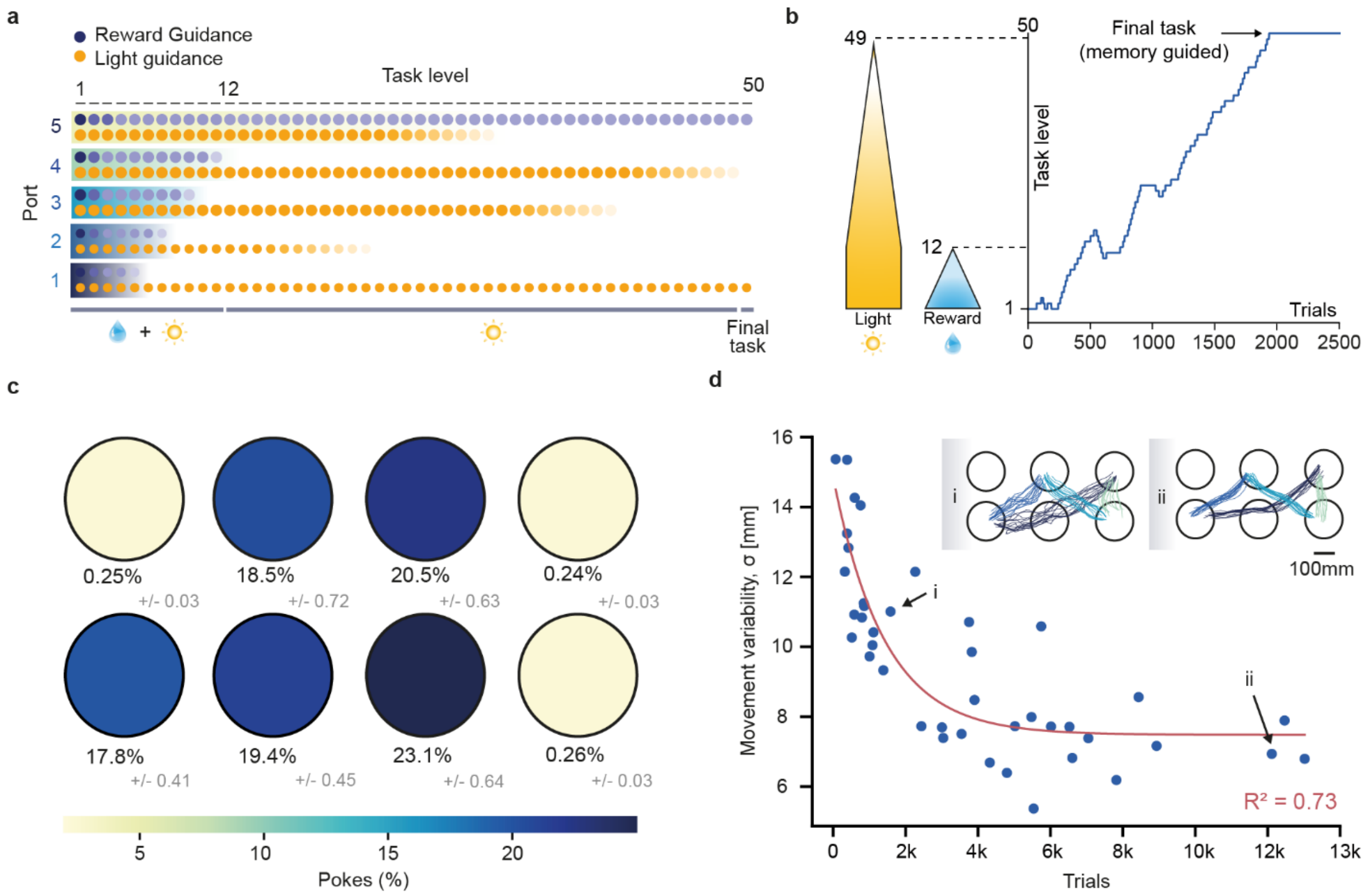


***Supplementary Fig. 1: Quantification for learning the sequence task.***

***a.*** *Schematic showing light guidance and reward delivery at each port in the task sequence for each training level.* ***b.*** *Example animal training level progression and regression learning curve. Reward and light guidance shut off points are denoted by dotted lines.* ***c.*** *Port poke occurrences (mean) for expert animals (trials 3000 to 3500, n = 33, +/- SEM).* ***d.*** *Average movement variability (standard deviation from average trajectory) across all subsequence task movements for mice at different levels of task experience (n = 8 mice, number of trials is total completed prior the tracking session, tracking point was centre of the head. Curve fit by non-linear least squares, two-sided F-test; ***p < 0.001, $R^2$ = 0.73). Inset: example tracking data showing subsequence trajectories from a novice (i) and an expert mouse (ii).*

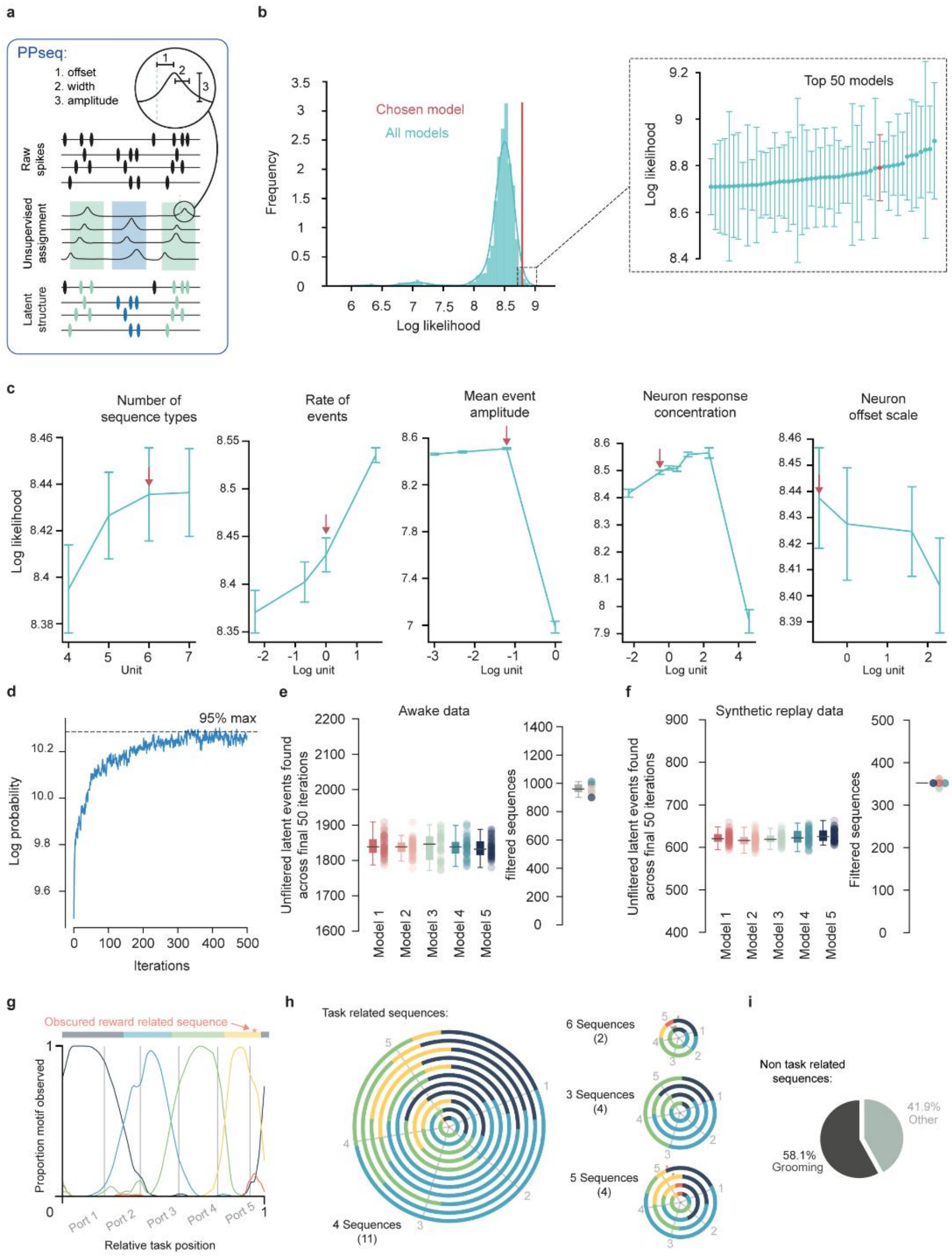


***Supplementary Fig. 2: Unsupervised labelling of neural sequences during task activity.***

***a.** Schematic diagram outlining unsupervised detection and labelling of latent neural structure from raw spikes by PP-Seq. The model takes raw spikes, infers latent causal events which can explain repeating sequential structures within the data, then labels spikes which contribute to those events parametrised by three features of neural firing; offset, amplitude and width. **b.** Histogram showing loss values for every combination of hyperparameters (models) tested in the grid search. The chosen model is indicated by the red line. Chosen model was hand selected from the top 20 models based on qualitative scoring of the spike labelling output. Loss values and error (SEM) for the top 50 models from the search, chosen model is showing in red. **c.** Loss values as a function of hyperparameter value for each varied parameter. Error bars are SEM loss for models in which the parameter of interest was fixed, and the other 5 variable hyperparameters were swept across the full range of tested values (models n = 1508). Red arrows show the value chosen. **d.** Log-likelihood over Gibbs samples. **e.** Left: number of identified latent events in the final 50 iterations for 5 independent model fits for real spike data (600s) taken from awake task related activity. Right: number of filtered task-fitted sequences identified from spike labelling for each independent model. **f.** Same as panel e, but for independently fitted awake models applied in replay detection mode to ground truth replay data (see methods). **g.** Left: relative sequence incidence curves across standardised task space for an example recording session. Grey lines indicate respective task port locations across standardised space. Curves are flattened into representation of sequence incidence across task space.*

| AP (mm) | ML (mm) | DV (mm) |
|---|---|---|
| -1.22 | +/- 0.75 | -1.80 |
| -1.58 | +/- 1.54 | -1.87 |
| -1.58 | +/- 0.81 | -2.06 |
| -1.58 | +/- 0.81 | -1.66 |
| -2.18 | +/- 0.90 | -1.83 |
| -2.18 | +/- 1.80 | -2.08 |
| -2.18 | +/- 1.80 | -1.58 |
| -2.18 | +/- 2.54 | -2.59 |
| -2.18 | +/- 2.54 | -2.09 |
| -2.80 | +/- 1.57 | -2.03 |
| -2.80 | +/- 1.57 | -1.53 |
| -2.80 | +/- 2.43 | -2.40 |
| -2.80 | +/- 2.43 | -1.90 |
| -2.80 | +/- 3.17 | -4.04 |
| -2.80 | +/- 3.17 | -3.54 |
| -2.80 | +/- 3.17 | -3.04 |
| -2.80 | +/- 3.17 | -2.54 |
| -2.80 | +/- 2.31 | -4.66 |
| -3.64 | +/- 3.02 | -4.33 |
| -3.64 | +/- 3.02 | -3.83 |
| -3.64 | +/- 3.02 | -3.33 |
| -3.64 | +/- 3.02 | -2.83 |
| -3.64 | +/- 2.53 | -3.90 |
| -3.64 | +/- 2.53 | -3.40 |
| -3.64 | +/- 2.53 | -2.90 |
| -3.64 | +/- 2.53 | -2.40 |

***Supplementary Table. 1: Injection locations for hippocampal lesions.***

| Hyperparameter | Meaning | Value | Chosen how |
|---|---|---|---|
| $\eta$ | Number of seq/motif types | 6 | Grid search |
| $\Psi$ | Rate of events | $\underline{1} \cdot 0.2 \cdot \eta$ | Grid search |
| $\gamma$ | Event concentration | 1 | Fixed |
| $\mu_A$ | Mean event amplitude | $0.3 \cdot N \cdot P$ | Grid search |
| $\sigma^2_A$ | Event amplitude standard deviation | $10 \cdot \mu_A$ | Fixed |
| $\varphi$ | Neuron response concentration | 0.6 | Grid search |
| $\nu$ | Neuron response width prior | 0.5 | Fixed |
| $\sigma$ | Neuron response width scale | 1 | Fixed |
| $\kappa$ | Neuron offset scale | 0.5 | Grid search |
| $\mu_0$ | Mean background spike rate | $0.3 \cdot N \cdot P$ | Fixed |
| $\sigma^2_0$ | Background spike rate variance | $(0.3 \cdot N \cdot P)^2$ | Fixed |
| $\gamma_0$ | Background firing rate concentration | 0.3 | Fixed |

N = number of neurons, P = average firing rate.

***Supplementary Table. 2: PP-Seq model hyperparameters***
